# Climatically controlled reproduction drives interannual growth variability in a temperate tree species

**DOI:** 10.1111/ele.13158

**Published:** 2018-09-19

**Authors:** Andrew J. Hacket‐Pain, Davide Ascoli, Giorgio Vacchiano, Franco Biondi, Liam Cavin, Marco Conedera, Igor Drobyshev, Isabel Dorado Liñán, Andrew D. Friend, Michael Grabner, Claudia Hartl, Juergen Kreyling, François Lebourgeois, Tom Levanič, Annette Menzel, Ernst van der Maaten, Marieke van der Maaten‐Theunissen, Lena Muffler, Renzo Motta, Catalin‐Constantin Roibu, Ionel Popa, Tobias Scharnweber, Robert Weigel, Martin Wilmking, Christian S. Zang

**Affiliations:** ^1^ Department of Geography and Planning School of Environmental Sciences University of Liverpool Liverpool UK; ^2^ Dipartimento di Agraria University of Naples Federico II via Università 100 80055 Portici (NA) Italy; ^3^ DISAA Università degli Studi di Milano via Celoria 2 20133 Milano Italy; ^4^ DendroLab Department of Natural Resources and Environmental Science University of Nevada Reno NV 89509 USA; ^5^ Biological and Environmental Sciences University of Stirling Stirling FK9 4LA UK; ^6^ Swiss Federal Institute for Forest, Snow, and Landscape Research WSL a Ramél 18 CH‐6953 Cadenazzo Switzerland; ^7^ Southern Swedish Forest Research Centre Swedish University of Agricultural Sciences P.O. Box 49 230 53 Alnarp Sweden; ^8^ Institut de recherche sur les forêts Université du Québec en Abitibi‐Témiscamingue 445 boulevard de l’ Université, Rouyn‐Noranda QC J9X 5E4 Canada; ^9^ Forest Research Centre, (INIA‐CIFOR) Ctra. La Coruñna km. 7.5 28040 Madrid Spain; ^10^ Department of Geography University of Cambridge Cambridge UK; ^11^ University of Natural Resources and Life Science – BOKU Vienna Austria; ^12^ Department of Geography Johannes Gutenberg‐University Johann‐Joachim‐Becher‐Weg 21 55128 Mainz Germany; ^13^ Institute of Botany and Landscape Ecology University of Greifswald 17489 Greifswald Germany; ^14^ Université de Lorraine AgroParisTech, INRA, UMR Silva 14 rue Girardet 54000 Nancy France; ^15^ Slovenian Forestry Institute Večna pot 2 SI‐1000 Ljubljana Slovenia; ^16^ TUM School of Life Sciences Professorship of Ecoclimatology Technical University of Munich Hans‐Carl‐von‐Carlowitz‐Platz 2 85354 Freising Germany; ^17^ Institute for Advanced Study Technical University of Munich Lichtenbergstraße 2 a 85748 Garching Germany; ^18^ Forest Growth and Woody Biomass Production TU Dresden, Pienner Str. 8 01737 Tharandt Germany; ^19^ DISAFA University of Turin Largo Braccini 2 10095 Grugliasco (TO) Italy; ^20^ Forest Biometrics Laboratory University “Stefan cel Mare” of Suceava Suceava Romania; ^21^ National Research and Development Institute in Forestry Marin Drăcea, Calea Bucovinei 73bis Campulung Moldovenesc Romania; ^22^ TUM School of Life Sciences Technical University of Munich Hans‐Carl‐von‐Carlowitz‐Platz 2 85354 Freising Germany

**Keywords:** Dendrochronology, drought, European beech, *Fagus sylvatica*, forest growth, masting, path analysis, SEM, structural equation modelling, trade‐off

## Abstract

Climatically controlled allocation to reproduction is a key mechanism by which climate influences tree growth and may explain lagged correlations between climate and growth. We used continent‐wide datasets of tree‐ring chronologies and annual reproductive effort in *Fagus sylvatica* from 1901 to 2015 to characterise relationships between climate, reproduction and growth. Results highlight that variable allocation to reproduction is a key factor for growth in this species, and that high reproductive effort (‘mast years’) is associated with stem growth reduction. Additionally, high reproductive effort is associated with previous summer temperature, creating lagged climate effects on growth. Consequently, understanding growth variability in forest ecosystems requires the incorporation of reproduction, which can be highly variable. Our results suggest that future response of growth dynamics to climate change in this species will be strongly influenced by the response of reproduction.

## Introduction

Tree growth and reproduction are key controls on the dynamics of forest ecosystems at a range of timescales, including their response to ongoing climate change. Both growth and reproduction are influenced by climate and resource availability. This makes them related, inducing growth‐reproduction trade‐offs in many species (Thomas 2011). Growing‐season climate influences growth via physiological processes including leaf phenology, photosynthesis and xylogenesis (e.g. Leuschner *et al*. 2001; Breda *et al*. 2006). However, the direction, duration, and timing of climate relationships with growth are not always consistent across space and time, and the processes accounting for the observed relationships are poorly understood, limiting our ability to predict future changes in tree growth (Babst *et al*. 2013; Guillemot *et al*. 2017; Peltier *et al*. 2018). A major source of such uncertainty are the processes that cause lagged effects of climate on growth (Piovesan *et al*. 2005; Hacket‐Pain *et al*. 2016). However, lagged effects of climate on growth are not well reproduced by vegetation models (Babst *et al*. 2013; Rammig *et al*. 2015). Despite recognition that they can be key drivers of tree growth responses to climate change (‘ecological memory’), the processes responsible are poorly understood (Ogle *et al*. 2015; Peltier *et al*. 2018). In some cases, lagged correlations can result from lagged effects within the physical environment (Woodhouse 2003). Alternatively, they have been attributed to ‘carry‐over’ or ‘legacy’ impacts within trees after unfavourable years (Anderegg *et al*. 2015). For example the depletion of internal carbohydrate reserves (Galiano *et al*. 2011) may reduce growth the following year (Skomarkova *et al*. 2006), although this has been difficult to demonstrate empirically, in part due to complexities of linking tree‐level resources and growth (Mund *et al*. 2010; Richardson *et al*. 2013). Additionally, leaf area index, xylem conductivity or fine root dynamic responses to climate may influence growth in subsequent years, creating mechanisms for lagged correlations between climate and growth (e.g. Breda *et al*. 2006; Galiano *et al*. 2011).

Allocation to reproduction is a key functional trait of plants, and varies both at interannual timescales, and across tree lifespan (Thomas 2011; Muller‐Haubold *et al*. 2013; Allen *et al*. 2014). Variation in reproductive effort is strongly influenced by climate, especially in species that display ‘masting’, i.e. highly variable interannual seed production synchronised among individuals and populations (Pearse *et al*. 2016; Vacchiano *et al*. 2017). Trade‐offs between growth and reproduction have been repeatedly observed, and can indirectly affect climate‐growth relationships, causing the emergence of lagged climate effects (Piovesan & Schirone 2000; Hacket‐Pain *et al*. 2015). Additionally, the strength of growth‐reproduction trade‐offs varies with abiotic stresses such as summer drought (Sletvold & Agren 2015; Hacket‐Pain *et al*. 2017), so the magnitude of growth reductions associated with investment in reproduction is also dependent on climate.

**Figure 1:**
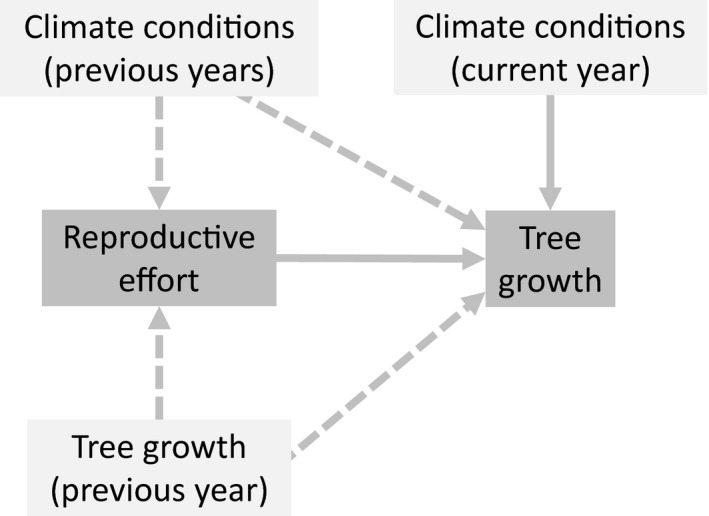
Theoretical model linking climate conditions across multiple years, tree reproductive effort and tree growth. Dashed lines indicate effects operating across years.

Here, we use a masting tree species (*Fagus sylvatica* L.) to investigate the interplay of climate, reproduction, and tree growth. We hypothesise that climate drives both resource availability and its allocation, including via lagged effects (Fig. 1). This implies that climatically controlled allocation to reproduction may be an important additional mechanism by which climate influences interannual variation in tree growth. Testing this hypothesis will improve our ability to understand and predict responses of trees to climate change (Selas *et al*. 2002; Drobyshev *et al*. 2010; Davi *et al*. 2016). We show that temperature and precipitation influence growth in this species both directly, and indirectly through controls on resource allocation to reproduction. Allocation to reproduction is a key driver of growth, and due to its predominant dependence on previous summers’ temperature, it is responsible for creating lagged climate effects on growth. Consequently, we argue that including variable resource allocation in models of tree growth will improve their ability to reproduce observed patterns of growth and improve predictions of future tree growth.

## Methods

### Data

**Figure 2:**
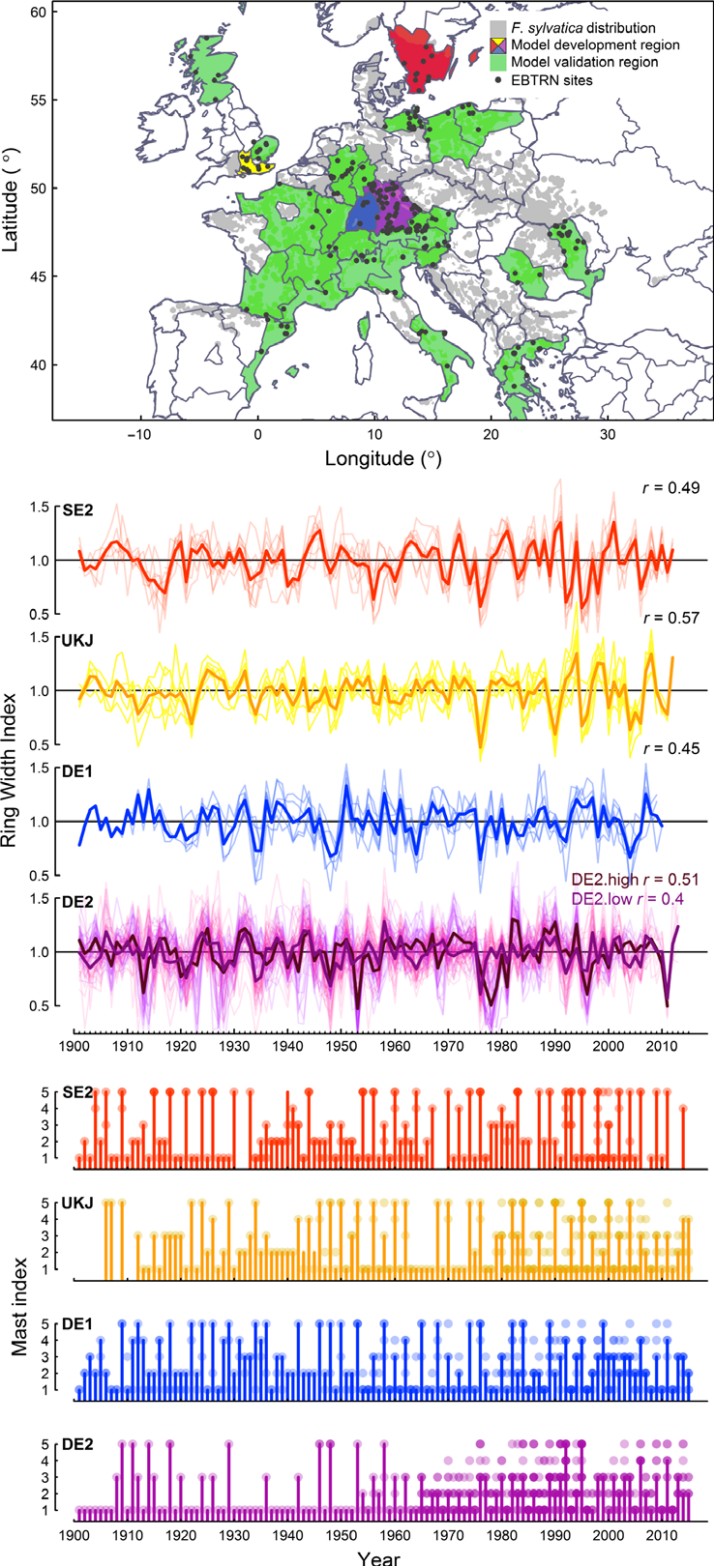
Study location and summary of data. (a) Study regions (NUTS‐1) including the geographic distribution of *Fagus sylvatica* (EURFORGEN 2009), and locations of individual RWI chronologies. (b) Ring‐width index chronologies for each region. Individual chronologies plotted in pale colours, and the mean regional chronology in dark colours. *r* represents the mean correlation between sites in each regional chronology. For DE2, cluster analysis revealed two distinct groups of chronologies, which correspond to high (paler purple) and low (darker purple) elevation (see Appendix B) (c) Annual reproductive effort *(RE)* (1‐2‐3 = non‐mast year; 4–5 = mast year) of *Fagus sylvatica* in each region. Individual records are plotted as points (colour intensity represents the number of records in a class), with the modal values plotted as bars.

Tree‐ring widths were used to characterise tree growth, with data taken from a tree‐ring network covering the whole geographic distribution of *F. sylvatica* (Fig. 2) (Zang *et al*. 2018). The dataset used in this study includes 321 sites, and extends from southern Scandinavia to the Mediterranean Basin, and from western Europe to the Balkans. Sites were selected to represent locally typical closed‐canopy *F. sylvatica* forest, and sampling was not designed to specifically target climatically stressed sites or individuals. Each site included a minimum of five trees. As our focus was to understand interannual variation in growth, low‐frequency ring‐width variation was removed using 32‐year spline detrending with a frequency cut‐off of 0.5 (Cook & Peters 1981), and individual trees were averaged to create mean site chronologies of ring‐width indices (*RWI*). Reproductive effort (*RE*) was characterised using a five‐class ordinal index of seed production (Ascoli *et al*. 2017a), with seed production chronologies for each NUTS‐1 (Nomenclature of Territorial Units for Statistics, see Appendices S1 and S2) region of Europe developed by Vacchiano *et al*. (2017). Ordinal data were reclassified to binary, comprising of ‘mast’ (category 4 and 5) and ‘non‐mast’ years (category 1, 2 and 3). This approach was designed to maintain linear relationships and reduce the degrees of freedom in the models. *RWI* series from individual sites were further averaged to create regional NUTS‐1 growth chronologies (Fig. 2), with correlations between sites in each NUTS‐1 checked to ensure growth synchrony within each region (Appendix S3). The number of individual site chronologies contributing to each mean NUTS‐1 chronology varied from 3 to 41**.** Data for monthly mean maximum temperature (*MAX*) and monthly total precipitation (*PRE*) were obtained from the CRU TS 3.23 gridded dataset (Harris *et al*. 2014). Regional climate time‐series were calculated by averaging pixel‐level climate data across NUTS‐1 using the cruts package (Taylor & Parida 2016) in R version 3.3.1 (R Development Core Team 2016). Homogeneity of climate within regions was checked by calculating the mean pairwise correlation between all individual grid cells in each region, and we checked that regional climate chronologies represented the climate of the sampled tree‐ring sites. Growing season conditions were represented by a 3‐month window (May–July, MJJ). While the time window corresponding to the strongest relationship with annual growth may vary between populations, previous studies have indicated this window captures the main signal for populations of *F. sylvatica* across Europe (Hacket‐Pain *et al*. 2016; Cavin & Jump 2017). Previous summer climate signals (t_‐1_ and t_‐2_) were represented using a 2‐month window (June–July, JJ), which acts as a consistent climate cue of mast years across Europe (Drobyshev *et al*. 2010; Muller‐Haubold *et al*. 2013; Vacchiano *et al*. 2017).

### Structural equation modelling (SEM)

We used SEM (path analysis) to examine relationships between climate variables, reproductive effort, and growth (Fig. 1), with analysis conducted at the NUTS‐1 regional level. SEM provides a tool to statistically test conceptual models with empirical datasets, including direct and indirect dependency structures (Fig. 1) (Grace 2006). In our initial model, growth was influenced directly by climate conditions in the growing season, which influence physiological processes including phenology, photosynthesis and xylogenesis (Leuschner *et al*. 2001; Breda *et al*. 2006). Links were also included to represent lagged effects of previous summer temperature on growth, which is commonly reported in this species (Piovesan *et al*. 2005). Additional climatic factors such as winter or spring temperature in the year of growth may be important locally, but are not consistently relevant across populations (Lebourgeois *et al*. 2005; Hacket‐Pain & Friend 2017), and so were not included in this analysis. Links were also included between current growth and annual reproductive effort (Muller‐Haubold *et al*. 2013; Hacket‐Pain *et al*. 2017), and between current‐ and prior‐year growth. Annual reproductive effort was influenced by previous summers’ climate and previous year growth (Drobyshev *et al*. 2010; Vacchiano *et al*. 2017). A full description and justification of our initial model is included in Appendix S4. Aggregated (NUTS‐1) data for *RWI*,* RE* and climate variables were tested for multivariate normality and multivariate outliers, using the MVN package in R (Korkmaz *et al*. 2014) (Appendix S5), and the linearity of bivariate relationships was checked by graphical plotting (Appendix S6). In SEM, the estimation of parameters aims to minimise the discrepancy between the observed covariance matrix and the covariance matrix implied by the hypothetical model (Grace 2006). SEMs were fitted using diagonally weighted least‐squares estimation (DWLS) in the R package lavaan (Rosseel 2012) in order to adjust for the categorical endogenous variables included in our data (*RE*). SEMs test the strength, sign and significance of relationships between variables. We used standardised path coefficients to represent these relationships, which can be interpreted as equivalent to partial correlation coefficients. For categorical variables we also plot the raw coefficients to aid interpretation (Grace & Bollen 2005). Indirect effects are estimated by multiplying coefficients along indirect pathways. The raw coefficients and *P*‐values are provided in Appendix S7.

### Model fitting and validation

Following the recommendation of Kline (2005), we focused the model development and fitting on regions with > 100 years of complete data, a threshold met by four regions: two in northern Europe (UKJ and SE2) and two in central Europe (DE1 and DE2) (Fig. 2). Initial analysis of the tree‐ring chronologies showed high within‐region synchrony between individual *RWI* chronologies in SE2, UKJ and DE1 (see Fig. 2), but lower synchrony in DE2. Cluster analysis revealed a strong dependence of *RWI* to elevation (see Appendix S8), therefore all analyses for DE2 were conducted using two mean chronologies (high and low elevation). Model development and fitting followed the two‐stage process recommended by Grace (2006). The first step focused on the concept of goodness‐of‐fit (GOF) and compared the specified model with the variance‐covariance matrix of observed data. This was essentially a test that no important links between variables were omitted. To estimate the GOF we used the *χ* ^2^ test (threshold value, *P* > 0.05), the Comparative Fit Index (CIF, threshold value > 0.9), and the Standardised Root Mean Square Residual (SRMSR) and Root Mean Square Error of Approximation (RMSEA) (threshold value < 0.1 and < 0.05 respectively) (Kline 2005; Grace 2006; Rosseel 2012). The second stage of evaluation investigated whether all links included in the model were supported by the data. Insignificant links (*P* > 0.05) were excluded from the model – but only if doing so did not reduce GOF (Grace 2006). As a final check, we compared models with and without the insignificant links using anova. This procedure was used to develop an optimal model for the prediction of *RE* and *RWI* based on climate and prior growth. To estimate confidence intervals around predicted *RWI* we randomly resampled model parameters 1000 times, assuming a normal distribution and using the estimated standard error for each parameter. A 95% confidence interval was based on the distribution of the set of 1000 predicted *RWI* (± 2 SD).

We then used independent regions with more limited data availability to validate our models using two approaches. First, we used the multi‐modelling approach (Rosseel 2012) to estimate a single model for all discrete regions used in the development of the optimal model structure. Then we ran this multi‐group model to predict *RWI* in 26 independent validation regions, based only on climate data, with *RE* predicted using previous summer temperatures. We used this model structure for further validation for eight of these 26 regions where we had at least 45 years of complete *RWI*,* RE* and climate data. Here we used the same optimal model structure, but the parameters were fitted individually in each region, allowing for local differences in sensitivity of growth and masting to climate. As these regions had not been used in model development and fitting, this was a form of model‐structure validation, testing the generality of our underlying assumptions of the controls of growth.

## Results

### Selection of optimal model linking climate, reproduction and tree growth

**Figure 3:**
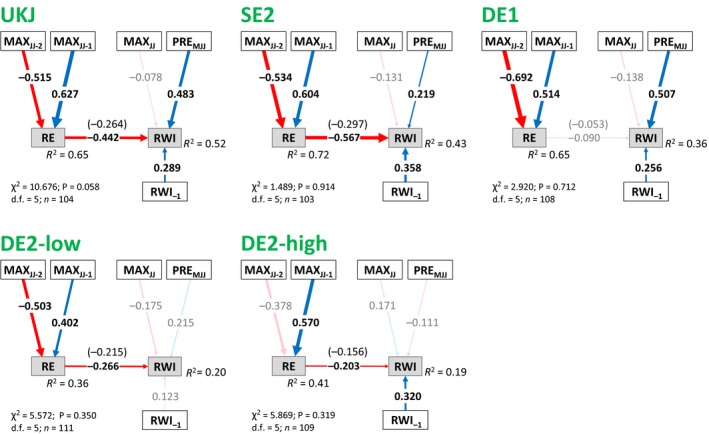
Structural Equation Models for model development and fitting regions, representing the effects of temperature and precipitation on radial growth, with indirect pathways involving the effects of allocation to reproduction (*RE*). Following mediation analysis, direct pathways from *MAX*_*JJ*__*‐1*_ and *MAX*_*JJ*__*‐2*_ to *RWI*, and from *RWI*_*‐1*_ to *RE*, have been removed. Blue and red arrows indicate positive and negative relationships respectively. Numbers on the arrows indicate the standardised path coefficients, with arrow thickness proportional to the coefficient strength. Coefficients in parenthesis refer to raw coefficients. Pale colours indicate non‐significant pathways (*P* < 0.05). The proportion of explained variance (*R* ^2^) for each endogenous variable is also shown.

Tree growth was significantly correlated with climate and reproductive effort in the five regions used for model development and fitting (Fig. 3 and Appendix S6). These models passed the GOF tests, showing that our proposed model structure linking climate, reproduction and growth was consistent with our datasets (Fig. 1). Growth was reduced in years of high reproductive effort, and was positively correlated with growing season precipitation (Fig. 3). However, not all the linkages in the original models were significant, and comparison of alternative models demonstrated that the effects of *MAX*_*JJ‐1*_ and *MAX*_*JJ‐2*_ on *RWI* could be adequately explained by indirect pathways involving *RE* (Appendices S9 and S10). In all five regions, a model that included only indirect effects of *MAX*_*JJ‐1*_ and *MAX*_*JJ‐2*_ via *RE* was statistically indistinguishable from a model that included both direct and indirect pathways, and significantly better than a model where previous summers’ temperatures could only influence growth directly (Appendix S10). This indicated that the indirect pathway via *RE* is the dominant (although not necessarily the exclusive) pathway for previous summers’ temperature influence on *RWI*. Consequently, as direct linkages between previous summers’ temperature and *RWI* were not supported by the data they were removed from the optimal model. The linkage between *RWI*_*‐1*_ and *RE* was also insignificant, and removing it had no effect on prediction of *RE. MAX*_*JJ*_ had no significant influence on growth in any of the five regions in the saturated models (Appendix S9). However, when models were optimised individually for each of the five regions (Appendix S11), *MAX*_*JJ*_ was retained in DE2‐high, becoming significant when *PRE*_*MJJ*_ was removed. We therefore decided to retain the linkage between *MAX*_*JJ*_ and *RWI* in the optimal models (Fig. 3). In conclusion, our optimal model structure predicted *RWI* on the basis of growing season climate (*PRE*_*MJJ*_ and *MAX*_*JJ*_), reproductive effort (*RE*) and previous year growth (*RWI*_*‐1*_), with additional indirect effects of previous summers’ temperature as *RE* was predicted on the basis of previous summers’ climate (*MAX*_*JJ‐1*_ and *MAX*_*JJ‐2*_).

**Figure 4:**
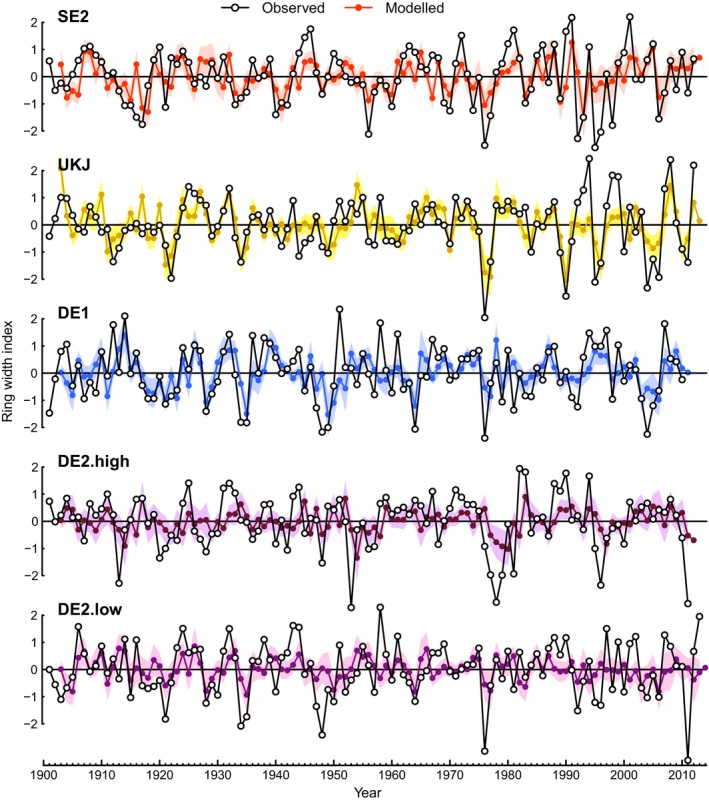
Comparison of observed and predicted *RWI* for model development regions (models described in Fig. 3). Shading represents 95% confidence interval for model predictions. Note that *RWI* is modelled as a function of *PRE*_*MJJ*_,*MAX*_*JJ*_ *,* and *RWI*_*‐1*_ *, and* predicted *RE* (predicted from *MAX*_*JJ*__*‐1*_ and *MAX*_*JJ*__*‐2*_) – i.e. observed *RE* is not used to predict *RWI*.

The optimal models explained a higher proportion of observed variance in *RWI* in northern Europe (*R* ^2^ = 55% and 43% in UKJ and SE2 respectively). Interpretation of coefficients is not straightforward in models with categorical variables (Grace & Bollen 2005), but in SE2 the raw coefficient for *RE*, which represents the change in *RWI* in a mast year (i.e. *RE *= 1), was greater than the standardised coefficient for *MAX*_*JJ*_ or *PRE*_*MJJ*_ *,* which represents the change in *RWI* for a ± 1 SD change in these climate variables. As a mast year occurred on average every 3 years in SE2 (Fig. 2), we suggest that *RE* was the variable with the strongest influence on *RWI* in this region. In UKJ, *RE* was also an important control on growth, but *PRE*_*MJJ*_ had an additional strong influence. In DE1, the model explained 36% of the variance in *RWI*, and *PRE*_*MJJ*_ was the strongest influence on growth (*RE* was insignificant). In DE2‐low and DE2‐high the models had lower explanatory power, and *RE* was the only significant linkage with *RWI*. In SE2, UKJ and DE1, the models reproduced observed patterns of *RWI* successfully, including multi‐year growth reductions (Fig. 4). However, fitted models for all regions failed to consistently reproduce the magnitude of growth reductions in years with the narrowest observed rings (Appendix S12). Consequently, the models did not adequately capture an observed increase in interannual growth variability during recent decades in UKJ and SE2.

There were differences in the relative importance of different pathways between regions (pathway coefficients) (Fig. 3). *RE* had a significant negative impact on growth in all regions except DE1, so that high *RE* (a mast year) resulted in reduced growth. Generally, summer precipitation (*PRE*_*MJJ*_) had a significant positive influence on growth, but this relationship was weaker in the most northerly region, SE2 and DE2‐low, and was insignificant in the high elevation region DE‐high. Summer temperature in the year of growth (*MAX*_*JJ*_) had no significant impact on growth in any of the study regions. While always insignificant, it had a weak negative influence on growth in all chronologies except DE2‐high, where the relationship was positive.

Consequently, in SE2 the main climate influence on radial growth was an indirect effect of temperature during the two summers prior to the year of growth, via *RE* (masting); i.e. the indirect influence of *MAX*_*JJ‐1*_ and *MAX*_*JJ‐2*_ were both greater than the direct influence of either *PRE*_*MJJ*_ or *MAX*_*JJ*_ (Fig. 3). In UKJ the influence of growing season precipitation (*PRE*_*MJJ*_) was stronger than in SE2, but previous summers’ temperature, through their influence on masting, also had a strong impact on growth. Radial growth in the previous year had a significant positive influence on growth (i.e. positive autocorrelation) in all five models.

### Model validation

A multi‐group model was fitted using the optimal model structure (Fig. 3), although restricted to UKJ, SE2 and DE2‐low. DE2‐high was excluded from the multi‐group model due to opposite influences of *PRE*_*MJJ*_ and *MAX*_*JJ*_ on *RW*I, and DE1 was excluded due to the insignificant link between *RE* and *RW*I. Including either of these regions in the multi‐group resulted in the model failing the GOF tests. The resulting multi‐group model was consistent with the results of the individual models (Appendix S13). The model captured the key features of growth in many of the 26 independent validation chronologies from across Europe for the period 1951–2015 (Fig. 6), including individual and multi‐year growth depressions, although the magnitude of these growth depressions was not reproduced by the models (Appendices S12 and S14). While correlations were low in some regions, it should be noted that errors in the prediction of *RE* propagate into the prediction of RWI. Indeed, in several of the regions where the model performed poorly, this was associated with lower model skill at predicting observed *RE*.

**Figure 5:**
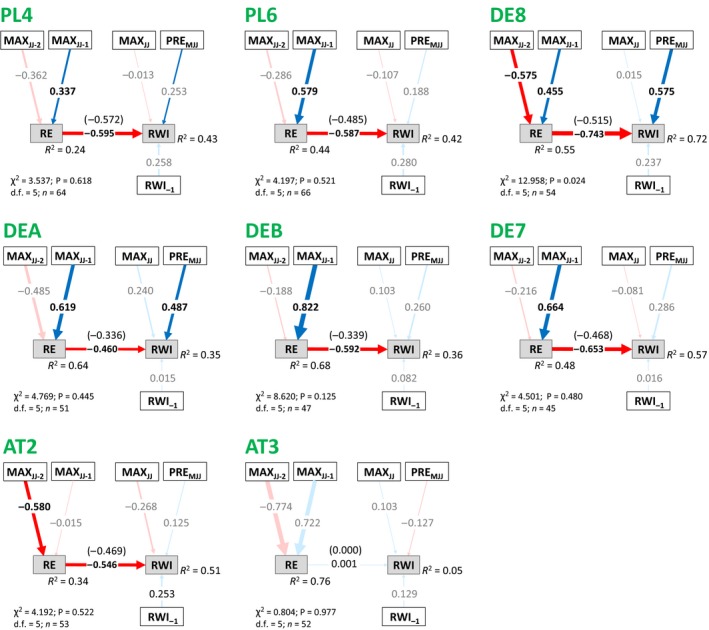
Model in Figure 3 fitted individually to each of an additional eight validation regions with ≥ 45 complete observations. Blue and red arrows indicate positive and negative relationships respectively. Numbers on the arrows indicate the standardised path coefficients, with arrow thickness proportional to the coefficient strength. Coefficients in parenthesis refer to raw coefficients. Pale colours indicate non‐significant pathways (*P* < 0.05). The proportion of explained variance (*R* ^2^) for each endogenous variable is also shown.

Then, we fitted the model structure in Fig. 3 individually to eight regions that had not been used in the model development due to smaller sample sizes. The reduced number of linkages in the optimal model allowed model fitting for regions with ≥ 45 years of data (Kline 2005) These individually fitted models (Fig. 5) supported the results of the optimal models for the five model development regions (Fig. 3), demonstrating that *RE* and *PRE*_*MJJ*_ are major drivers of growth. Seven of the eight models passed the GOF tests (DE8 failed, and was not included further). *RE* had a significant negative influence on *RWI* in six of these seven remaining validation models (in addition to the significant negative influence in four of the five original models), and was the largest direct or indirect effect on growth in five regions. *PRE*_*MJJ*_ had a significant positive influence on growth in one region (DEA), and was positive but insignificant in all other regions except AT3. The influence of *MAX*_*JJ*_ was always insignificant, and was negative in all regions except DEB and AT3.

## Discussion

Across a wide geographical region, a simple model structure with direct influences of summer precipitation and temperature, a negative impact of reproductive effort and an autoregressive term was consistent with observed data (12 of 13 models passed the GOF tests), and explained a high proportion of observed variation in growth in most regions. While the coefficients associated with each linkage showed regional variability (Figs 3 and 5), our results show that interannual variation in growth in *F. sylvatica* can be adequately explained by a framework of direct growing‐season climate effects and climatically driven variation in annual allocation to reproduction. Importantly, in eight of twelve individually fitted models (Figs 3 and 5), the main factor driving interannual variation in ring width was allocation to reproduction (*RE*) (Figs 3 and 5). The influence of climate on growth varied in importance (and direction) between regions, but *RE* had a consistently negative effect on growth; i.e. mast years reduced growth. Importantly, the *RE* influence was consistent across the species’ geographic distribution, generalising the results of regionally focused studies (Drobyshev *et al*. 2010; Muller‐Haubold *et al*. 2013; Hacket‐Pain *et al*. 2017). In our analysis, in seven of the twelve individually fitted models, high *RE* (a mast year) was associated with a larger growth reduction than a dry summer with precipitation one standard deviation below the mean (and in an additional region the coefficients were equal in magnitude). As years of high *RE* usually occur more frequently than dry or warm summers, this implies that *RE* has a greater effect on growth than these climate variables. We note that the window used for growing season climate influences on growth was fixed for all regions, and other climatic influences on growth were not included in our analysis, such as late frost events (Príncipe *et al*. 2017). If the climate window were optimised for individual regions, the relative importance of direct climate influences and *RE* may change and the overall variance explained by the models would increase (Lebourgeois *et al*. 2005; Hartl‐Meier *et al*. 2014). Weaker relationships between intraregion *RE* and *RWI* were found in some regions, which may result from variation in the strength of growth‐reproduction trade‐offs among populations (Sletvold & Agren 2015), perhaps due to differences in non‐structural carbohydrate (NSC) storage. However, variation between regions could also be related to the data used to characterise reproductive effort (Ascoli *et al*. 2017a). Intraregions *RE* correlations were generally high (Fig. 2 and Appendix S2), but fine‐scale variations in reproduction effort may not be captured by the data used in this study (Wohlgemuth *et al*. 2016). Nevertheless, our results provide strong evidence that allocation to reproduction has a consistent negative effect on the growth of *F. sylvatica*, and can explain a substantial part of the observed interannual variation in ring width (Drobyshev *et al*. 2010; Muller‐Haubold *et al*. 2013; Hacket‐Pain *et al*. 2017).

Furthermore, because *RE* was itself correlated with temperature from previous summers (Fig. 3, and see also Vacchiano *et al*. 2017), an important indirect temperature control on growth was revealed. *RWI* is frequently reported to be negatively correlated with previous summer temperatures (Babst *et al*. 2013; Hartl‐Meier *et al*. 2014; Hacket‐Pain *et al*. 2016), but comparing alternative models indicated that for our data, the most parsimonious model included only indirect pathways through *RE* (Figs 3 and 5). In other words, adding direct links between previous summer temperature and growth did not improve the model skill at reproducing *RWI*. We interpret this to suggest that lagged correlations between growth and previous years temperature are largely a consequence of the interplay with reproduction (masting) (Hacket‐Pain *et al*. 2015), rather than resulting from other ‘carry‐over’ effects. Further evidence to support this conclusion comes from the consistent direction of the indirect temperature influences. While the coefficients associated with the direct *MAX*_*JJ*_ *‐RWI* linkage varied from positive to negative with elevation in central Europe, the influences of previous summers’ temperature through *RE* were consistently negative (Figs 3 and 5). This indicates that the lagged negative correlations between *RWI* and previous summer temperature (*MAX*_*JJ‐1*_) are not primarily driven by ‘carry‐over’ effects of hot summers on internal resources levels (e.g. NSC) (Guillemot *et al*. 2017), as the influence of growing season temperature (and precipitation) on growth switches sign, but the lagged effects do not. Furthermore, regions with weaker *RE*‐*RWI* relationships also had weaker bivariate correlations between *MAX*_*JJ‐1*_ and *RWI* (Fig. 3 and Appendix S6). However, we acknowledge that other mechanisms act over multiple years to create lagged effects on growth, including NSC dynamics, and needle, leaf and root dynamics (e.g. Fritts 1976). These climate‐dependent processes are also important controls of growth in *F. Sylvatica*, and may be the main drivers of lagged climate correlations in species that do not exhibit one or more of the key characteristics that underpin the climate‐reproduction‐growth interplay that we have explored here. Many tree species or populations do not have synchronised and highly variable investment in reproduction (masting) (Herrera *et al*. 1998)**,** show weak relationships between climate and reproductive effort (Vacchiano *et al*. 2017; Patterson & Knapp 2018), or do not exhibit a strong negative relationship between reproductive effort and radial growth (e.g. Lebourgeois *et al*. 2018; Patterson & Knapp 2018). For example interplay with reproduction seems unlikely to explain the lagged effects of drought on growth in non‐masting conifers growing in the southern and western North America (e.g. Anderegg *et al*. 2015; Peltier *et al*. 2018).

Consequently, the indirect influence of *MAX*_*JJ‐1*_ on growth (through *RE*) was an important climatic driver of growth, and in half of the regions (6/12) the total effect of *MAX*_*JJ‐1*_ on growth was greater than the direct influence of growing season climate (*MAX*_*JJ*_ or *PRE*_*MJJ*_). Furthermore, in this study we characterised *RE* using a binary measure of allocation to reproduction. Higher resolution data may reveal that the variation in *RWI* explained by reproductive effort is greater than we found here (Hacket‐Pain *et al*. 2017). It is also notable that alternative models that only included indirect pathways for the effects of *MAX*_*JJ‐1*_ and *MAX*_*JJ‐2*_ through *RE* captured observed variation in *RWI* more successfully than when only direct links were included (Appendix S10). This indicates that the ability of *MAX*_*JJ‐1*_ and *MAX*_*JJ‐2*_ to predict *RWI* is improved when they are combined to predict the probability of a year with high *RE* (i.e. a mast year). In other words, models that included climate effects through *RE* were better at predicting growth than alternative models that included direct lagged effects on growth (*MAX*_*JJ‐1*_ and *MAX*_*JJ‐2*_) but no influence of *RE* (Appendix S10).

Our results imply that adequately explaining observed variation in tree growth requires accounting for flexible allocation of resources, including reproduction, which is a major sink for carbohydrates and nutrients and is highly variable across years (Muller‐Haubold *et al*. 2013; Pearse *et al*. 2016). In particular, the effect of reproductive allocation on growth will be an important factor determining the response of growth to future climate changes. Some studies have reported increased investment in reproduction in recent decades (Allen *et al*. 2014), which may have negative effects on forest productivity over short and longer timescales, analogous to the effects of changes in the occurrence of insect outbreaks (Peters *et al*. 2017). Indeed, our results show that a major source of uncertainty in the prediction of future changes in tree growth may originate from uncertainty in the response of tree reproductive effort to climate change (Ascoli *et al*. 2017b; Pearse *et al*. 2017). The proximate drivers of variable seed production in *F. sylvatica* are still uncertain, but the positive correlation between previous summer temperature and seed production has been linked to floral primordia differentiation (Drobyshev *et al*. 2010; Vacchiano *et al*. 2017). The negative correlation with MAX_JJ‐2_ may be related to climate effects on resource accumulation (Pearse *et al*. 2016; Allen *et al*. 2017; Ascoli *et al*. 2017b).

Our simple models explain a high proportion of the observed variance in growth. In the twelve regions where models were fitted individually, the combination of growing season precipitation, temperature, previous summer's growth and *RE* could explain ≥ 35% of the observed variance in interannual growth in half of the regions (*R* ^2^ ≥ 50% in three of the twelve regions) (Figs 3 and 5). Generally, the lowest tree growth was associated with a combination of high *RE* and dry summers. While these terms were included in the final models, they did not fully reproduce the magnitude of observed growth reductions in years of extreme low growth (e.g. 1976). This may be due to nonlinear responses of growth to climate, particularly under climate extremes (Appendix S6), and to interactions, such as between low precipitation and/or high vapour pressure deficit and high temperature, or interactions between low precipitation and reproductive effort. A potential effect of tree age on allocation to reproduction was also not included in our model (Thomas 2011). Other climate factors that we did not include in our models may be important controls on growth locally (Piovesan & Schirone 2000; Skomarkova *et al*. 2006; Drobyshev *et al*. 2010). For example in mountain and upland regions late spring frost events occurring after leaf‐out are associated with narrow tree rings (Dittmar *et al*. 2006; Príncipe *et al*. 2017), and low growth in the DE2‐high chronology in 1927, 1928, 1953 and 2011 correspond to late frost events reported in the region by Dittmar *et al*. (2006) and Menzel *et al*. (2015).

**Figure 6:**
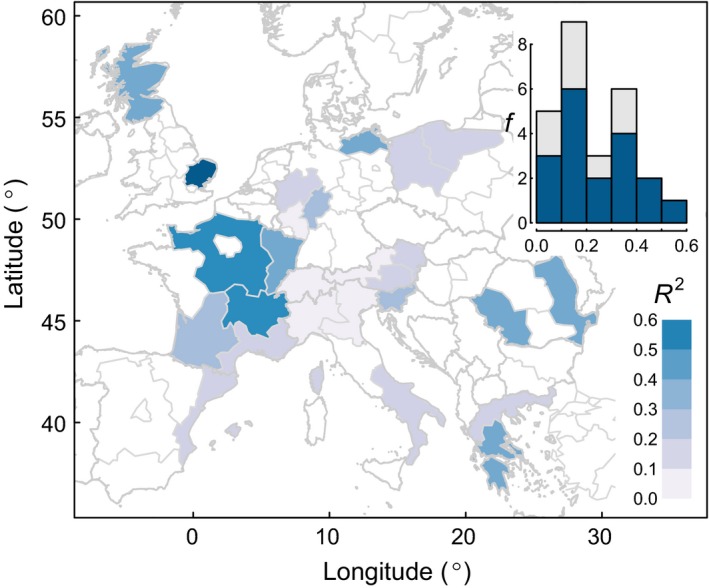
Comparison of predicted and observed tree ring chronologies from independent validation regions. *RWI* was predicted for each region using the multi‐group model. Note that in these models, *RE* was predicted using climate data, and predicted *RE* is then used in the model predicting *RWI*. The inset frequency plot shows the distribution of *R* ^2^, with light grey bars indicating regions where the regional RWI chronology shows low intraregion synchrony (mean correlation between sites < 0.3, see Appendix S3).

Our multi‐group model reproduced elements of the independent *RWI* series across the species’ geographic distribution, with higher correspondence between observed and predicted *RWI* in Romania, France and the UK (Fig. 6). As expected, the explained variance in these regions was lower than for the individually fitted models (Figs 3 and 5), as the multi‐group model used for predicting *RWI* in the independent datasets did not allow for geographic variation in the sensitivity of *RWI* to growing season climate or *RE*, which was observed in this and previous studies (Piovesan *et al*. 2005; Cavin & Jump 2017). For example the coefficient associated with summer precipitation was highest in westerly regions (UKJ, DE7, DE1), and was frequently insignificant in eastern regions (AT2, AT3, DE2, PL6). In particular, the model was less successful at predicting *RWI* in the Alpine region (Austria, Switzerland, northern Italy). It should be noted that interannual growth synchrony was low in many of these topographically complex regions (Appendix S3), indicating diverse drivers of growth or microclimatic variation in topographically complex regions (Hartl‐Meier *et al*. 2014; Hacket‐Pain & Friend 2017). Additionally, previous analysis has revealed that while broad‐scale climate cues of mast years are consistent between populations in *F. sylvatica* (Vacchiano *et al*. 2017), there are some differences in the seasonality and nature of cues between populations. Consequently, we would expect the multi‐group model to vary in its ability to accurately predict *RE*.

## Conclusions

We have found that climatically driven variation in reproductive effort is an important control on interannual growth variability in *F. sylvatica* and appears to be the dominant factor determining interannual radial growth variability in many populations. Additionally, the climatic control of reproductive effort creates indirect climate effects on growth, generating lagged correlations between summer temperature and growth. A similar interplay may be important in explaining antecedent climate effects on growth in other species (Anderegg *et al*. 2015; Peltier *et al*. 2018), but this remains to be fully tested. Importantly, this study also demonstrates that categorical and regional‐resolution data on mast years can provide useful information to untangle the interplay of climate, reproduction, and tree growth. These results have important implications for models of tree growth, including those that resolve annual ring width or simulate tree growth or NPP. Such models have tended to focus on the direct effects of growing season climate on growth, using approaches that range from phenomenological (e.g. Tolwinski‐Ward *et al*. 2011) to physiologically focused process‐based models (e.g. Friend & White 2000). Our results demonstrate that climatically controlled variation in allocation to reproduction is an important control on tree growth (at least for beech), and contribute to a growing body of research that indicates that variable resource allocation at a variety of timescales is a key factor influencing tree growth (Drobyshev *et al*. 2010; Thomas 2011; Muller‐Haubold *et al*. 2013; Hacket‐Pain *et al*. 2017). For example Guillemot *et al*. (2015) suggest that increased investment in reproduction with tree age could explain age‐related declines in stem biomass increment (see also Thomas 2011). Decadal variations in reproductive effort (e.g. frequency of ‘mast years’) (Drobyshev *et al*. 2014; Ascoli *et al*. 2017b) may have effects on growth trends analogous to the influence of cyclic insect outbreaks (Peters *et al*. 2017). Consequently, we argue that including variable resource allocation in models will increase the ability to reproduce observed variability in tree growth and growth‐climate relationships. It may also improve predictions of future changes in tree growth, which will at least in part be dependent on the response of reproduction to environmental change.

## Statement of authorship

AJHP conceived and designed the study, conducted analysis and wrote the manuscript. CZ contributed to study design, preliminary analysis and the manuscript preparation. GV and DA helped to conceive the study, prepared datasets and contributed to the manuscript preparation. ADF contributed to the initial development of the study concept. All authors contributed data and contributed to manuscript revision and editing.
