# Supplementary material for: Climatically controlled reproduction drives interannual growth variability in a temperate tree species
Source: Ecol Lett. 2018 Sep 19;21(12):1833–44. doi: 10.1111/ele.13158 (PMC6446945; doi:10.1111/ele.13158)

Appendix S1

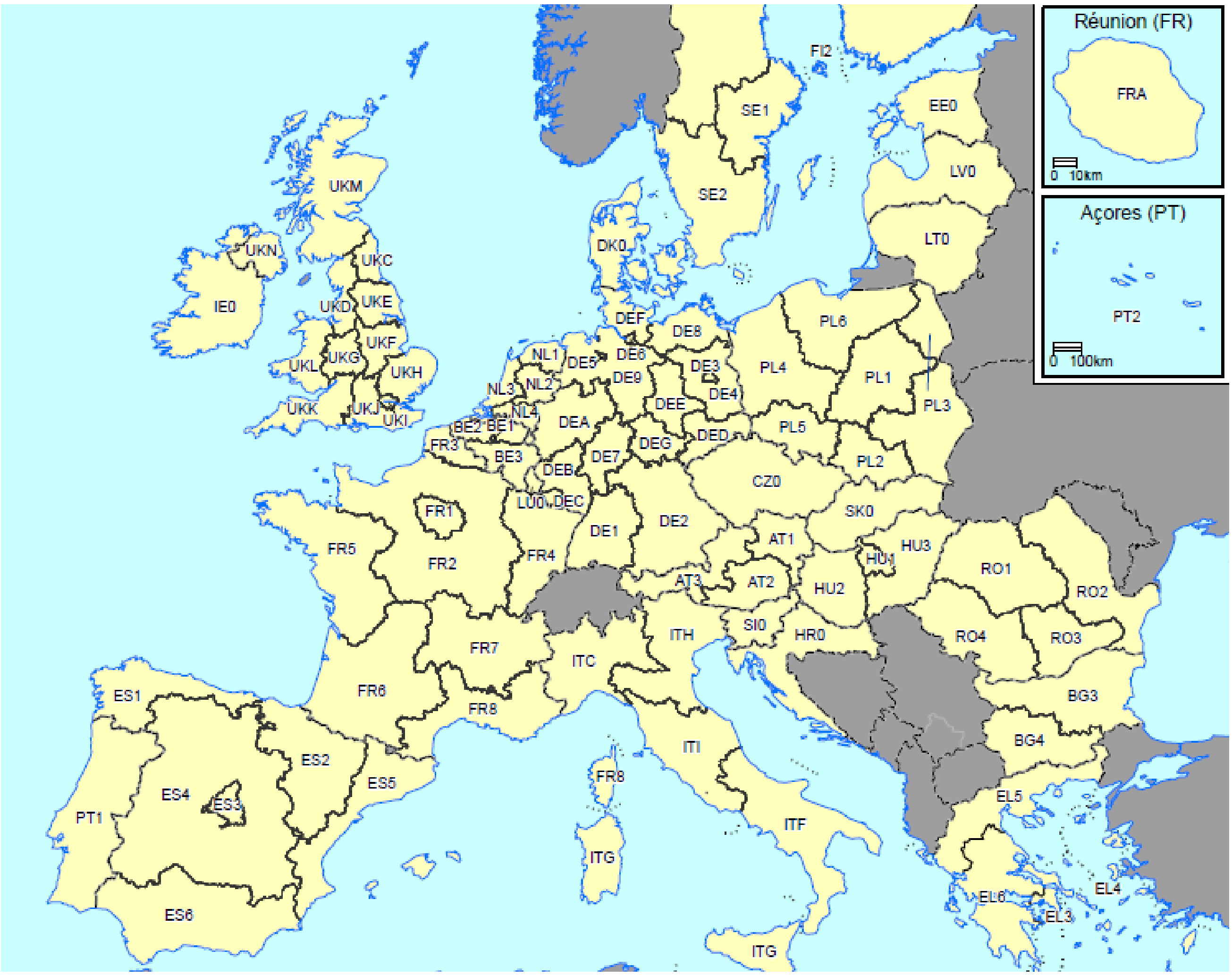

© EuroGeographics Association for the administrative boundaries. Cartography: Eurostat - GISCO, 2014

Map of European NUTS-1 regions (Nomenclature of Territorial Units for Statistics)

Appendix S2

NUTS-1	n	rho
AT2	5084	0.281
AT3	10857	0.193
DE1	314	0.692
DE2	1656	0.435
DE7	98	0.987
DE8	-	NA
DEA	-	NA
DEB	-	NA
PL4	670	0.818
PL6	225	0.598
SE2	159	0.950
UKJ	371	0.628

Intra-NUTS correlation of masting series for regions used in model development and fitting and model validation, representing synchrony of independent masting records from each region. Mean spearman rank correlation of all pairs of observations from the same region with >=7 years of overlap, with Hotelling correction for n<30 (see Vacchiano *et al*. 2017), for the period 1901-2016. No overlapping records exist for DE8, DEA and DEB.

Appendix S3

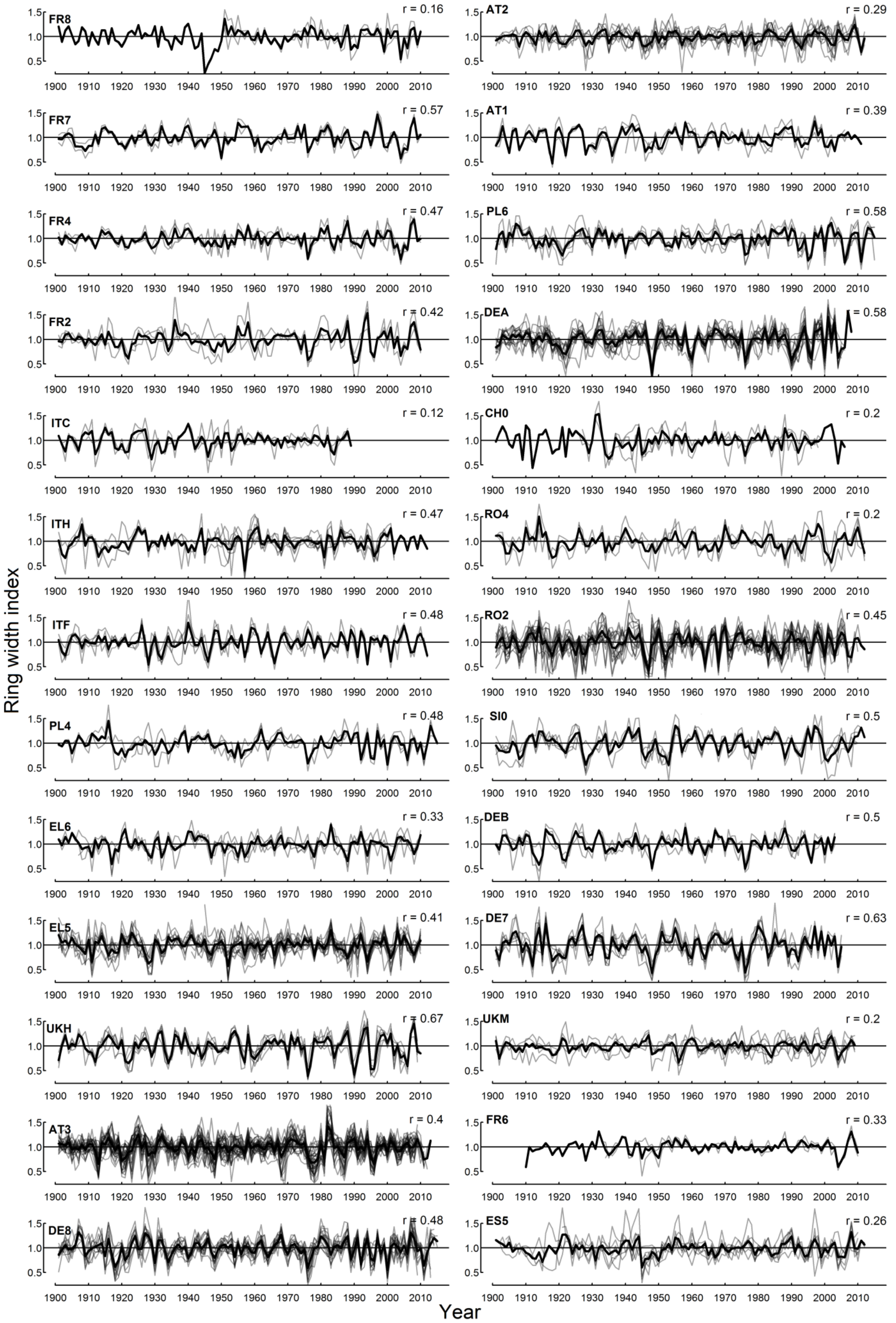

Individual site and region RWI chronologies for all regions in EBTRN with ≥3 individual sites, and the mean intra-site correlation

Number of contributing chronologies for each NUTS region

NUTS region	Number of sites	NUTS region	Number of sites
FR8	3	AT2	10
FR7	3	AT1	3
FR4	3	PL6	5
FR2	3	DEA	16
ITC	3	CH0	3
ITH	7	RO4	3
ITF	6	RO2	17
PL4	3	SI0	4
EL6	4	DEB	3
EL5	17	DE7	7
UKH	7	UKM	6
AT3	33	FR6	3
DE8	15	ES5	7

Appendix S4

Justification of the original theoretical model. See also Figure 1.

In our model, growth is influenced directly by climate conditions in the growing season (temperature and precipitation), which are known to influence physiological processes including leaf phenology, photosynthesis, and xylogenesis processes (e.g. Breda et al. 2006; Leuschner et al. 2001; Muller et al. 2011). Numerous studies have reported lagged correlations between tree-ring growth and previous summer climate conditions (Babst et al. 2013; Hacket-Pain et al. 2016; Piovesan et al. 2005), and so the model also includes links to represent these lagged effects. Growth is also influenced by prior-year growth, as shown by the typically high first-order autocorrelation present in tree–ring widths. This biological 'memory' may reflect the importance of stored carbohydrates on current increment, especially at the start of the growing season (Richardson et al. 2013; Skomarkova et al. 2006). Additional climatic factors may be important locally (Dittmar et al. 2003; Drobyshev et al. 2010; Piovesan and Schirone 2000, Hartl-Meier et al. 2014, 2015), but are not consistently relevant across populations (Hacket-Pain et al. 2016), and so are not included in this analysis. Growth is also influenced by annual reproductive effort.

Our model also includes linkages between reproductive effort and summer temperature in the two previous summers (Vacchiano et al. 2017), as well as a link to previous year growth, as some studies have indicated that increased investment in reproductive effort maybe associated with higher growth in the previous year (Drobyshev et al. 2010). Previous summers' temperature is the main driver of variation in seed production in *Fagus sylvatica* (Ascoli *et al.* 2017; Drobyshev *et al.* 2010; Vacchiano *et al*. 2017), so no linkage between current year climate and reproduction was included in our model.

Consequently, the model allows for direct effects of climate on growth, and indirect effects of previous years' climate through an effect on allocation to reproduction.

Appendix S5

Multivariate normality tests using the MVN package. All regions used for SEM fitting tested using three tests in the MVN package, plus graphic tests recommended in MVN vignette (MVN: An R Package for Assessing Multivariate Normality).

Where regions failed a test, and MV outliers were identified, the tests were re-applied on datasets with outliers removed. Visual inspection of the data using MVN tools indicates that all datasets were suitable for further analysis, and that any non-normality is minor.

p-values of various tests in MVN package. Red = highly significant, orange = marginally significant. Years in bold are highlighted as potential outliers in multiple regions.

	mariaTest (chi.skew)	mariaTest (chi.kurt)	hzTest	roystonTest	Multivariate outliers? (numbers = years, i.e. 76=1976)	Removing outliers = pass all tests?
UKJ	0.00603784	0.200999	0.1737328	0.4099062	None	
SE2	0.1858262	0.4119942	0.1646647	0.6187616	None	
DE1	0.8262608	0.4592369	0.2370057	0.3733476	None	
DE2.l	0.2237479	0.8159374	0.4033275	0.1177207	1948, 2006, **2007, 2011**,2013	NA
DE2.h	0.04136057	0.8022064	0.0645655	0.001358285	1953, 1978, **2011**	Yes
AT2	0.8246736	0.3149806	0.6652836	0.4614074	None	
AT3	0.9921031	0.4090627	0.8288373	0.1361042	None	
DE7	0.6498858	0.315142	0.1204587	0.1548945	None	
DE8	0.1608481	0.00737824	0.3659882	0.05480976	**1976**, 1987,1992**, 2007, 2011**	Yes
DEA	0.6863757	0.5708077	0.4833007	0.3675073	None	
DEB	0.1276757	0.5638072	0.1408494	0.03196274	**1976**, 1980, 2000	Yes
PL4	0.5943207	0.5677678	0.7808395	0.5204973	None	
PL6	0.1007822	0.7760077	0.0294171	0.08319151	1995, 1996, 2006, **2011**	Yes

Appendix S6

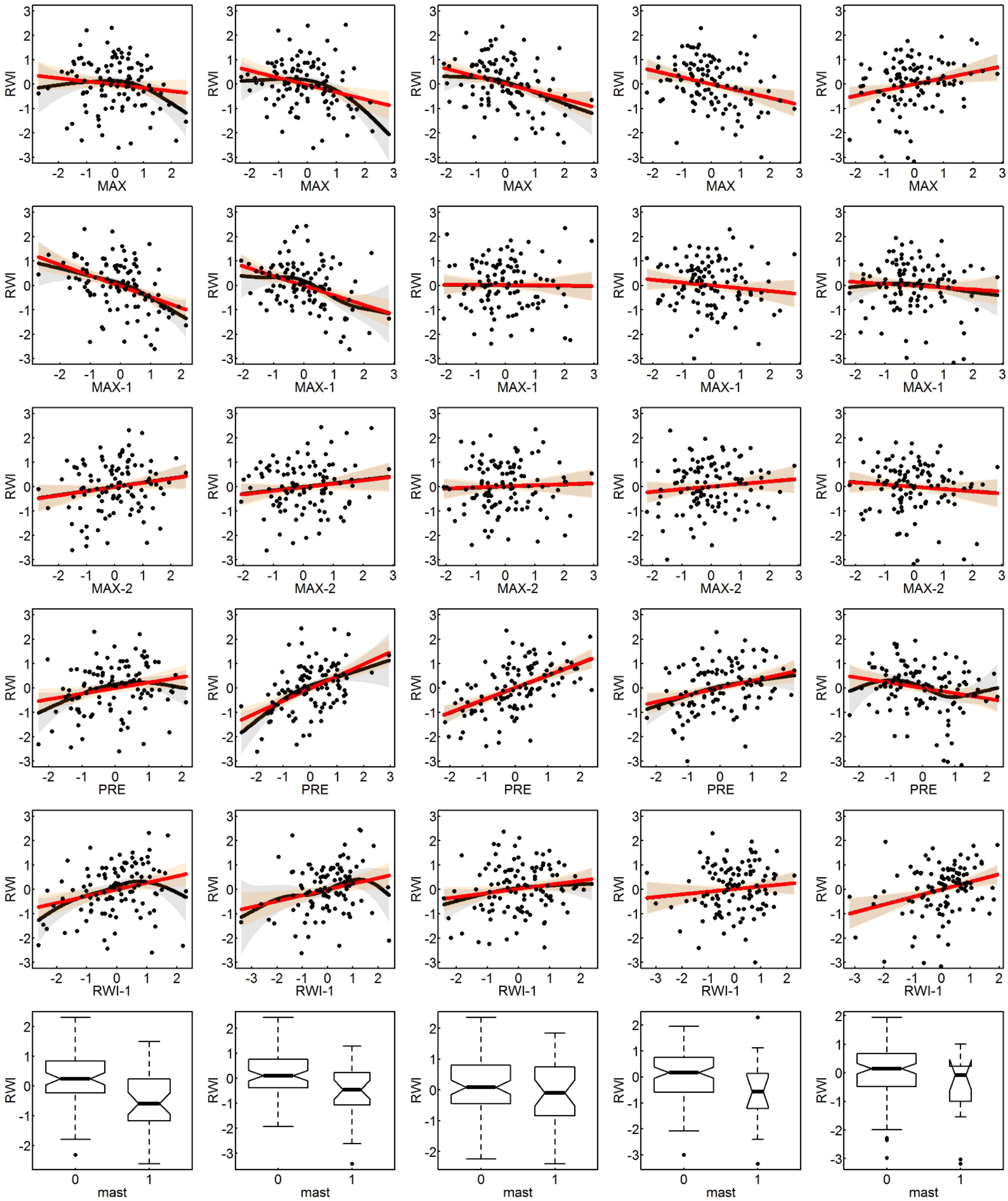

Biplots for SE2, UKJ, DE1, DE2-low, DE2-high. Linear models and GAMs are used to illustrate relationships between RWI and climate, previous years' growth (RWI$_{-1}$) and *RE* (mast years) (boxplots).

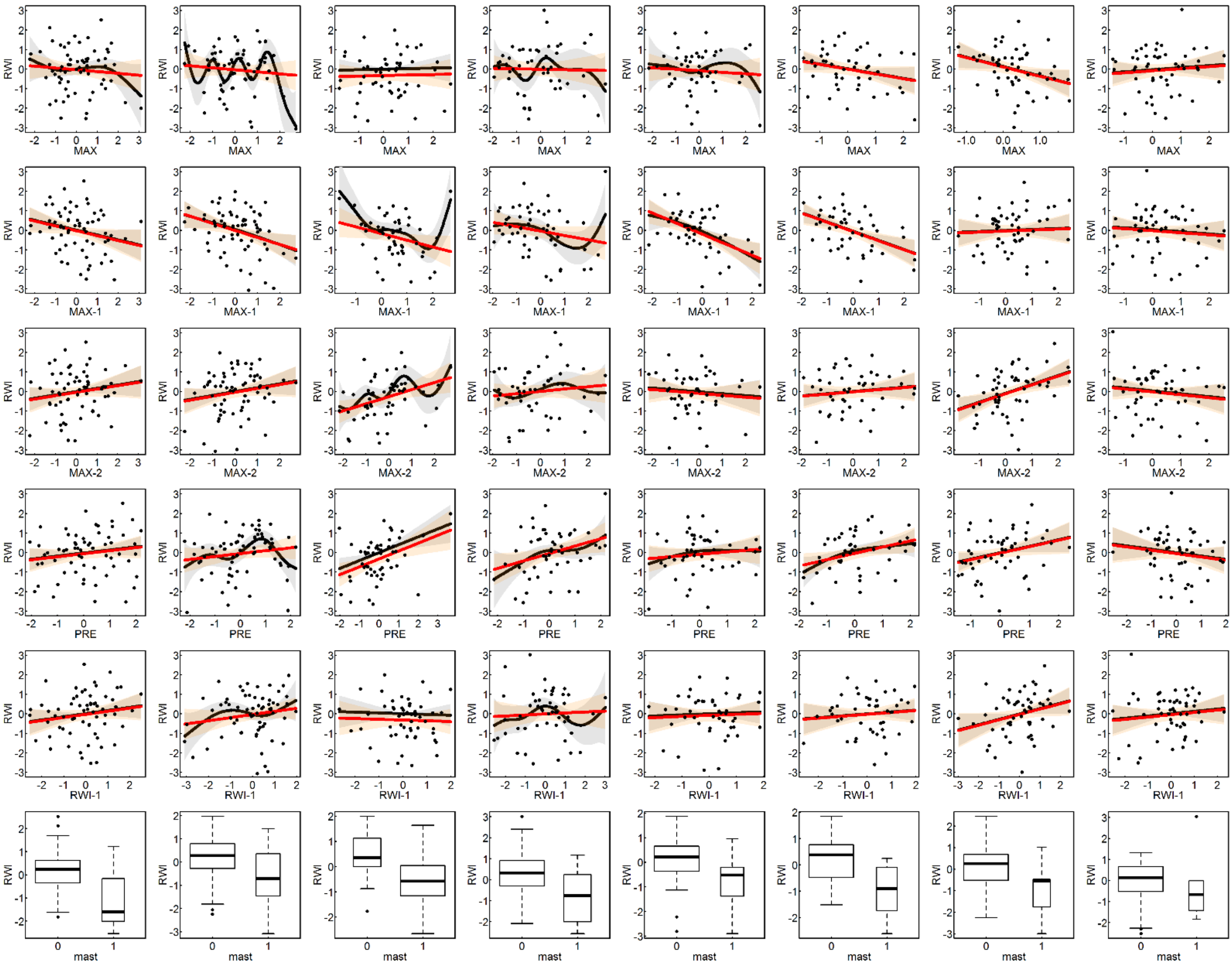

Biplots for PL4, PL6, DE8, DEA, DEB, DE7, AT2, AT. Linear models and GAMs are used to illustrate relationships between RWI and climate, previous years' growth (RWI_{-1}) and *RE* (mast years) (boxplots).

Appendix S7

Full model summaries for models reported in Figure 3

UKJ

```
                                                  Used       Total
  Number of observations                           104         115

  Estimator                                       DWLS
  Minimum Function Test Statistic               10.676
  Degrees of freedom                                 5
  P-value (Chi-square)                           0.058

Model test baseline model:

  Minimum Function Test Statistic              102.532
  Degrees of freedom                                11
  P-value                                        0.000

User model versus baseline model:

  Comparative Fit Index (CFI)                    0.938
  Tucker-Lewis Index (TLI)                       0.864

Root Mean Square Error of Approximation:

  RMSEA                                          0.105
  90 Percent Confidence Interval          0.000  0.193
  P-value RMSEA <= 0.05                          0.129

Standardized Root Mean Square Residual:

  SRMR                                           0.089

Weighted Root Mean Square Residual:

  WRMR                                           0.873

Parameter Estimates:

  Information                                 Expected
  Standard Errors                             Standard

Regressions:
                   Estimate  Std.Err  z-value  P(>|z|)   Std.lv  Std.all
  RWI ~
    PRE               0.495    0.097    5.082    0.000    0.495    0.483
    MAX              -0.079    0.080   -0.992    0.321   -0.079   -0.078
    mast             -0.264    0.064   -4.109    0.000   -0.264   -0.442
    RWI.lag1          0.292    0.074    3.934    0.000    0.292    0.289
  mast ~
    MAX.L             1.107    0.229    4.825    0.000    1.107    0.627
    MAX.LL           -0.878    0.217   -4.045    0.000   -0.878   -0.515

Intercepts:
                   Estimate  Std.Err  z-value  P(>|z|)   Std.lv  Std.all
   .RWI              -0.012    0.083   -0.151    0.880   -0.012   -0.012
   .mast              0.000                               0.000    0.000

Thresholds:
                   Estimate  Std.Err  z-value  P(>|z|)   Std.lv  Std.all
    mast|t1           1.074    0.247    4.343    0.000    1.074    0.634

Variances:
                   Estimate  Std.Err  z-value  P(>|z|)   Std.lv  Std.all
   .RWI               0.487    0.092    5.293    0.000    0.487    0.476
   .mast              1.000                               1.000    0.348

Scales y*:
                   Estimate  Std.Err  z-value  P(>|z|)   Std.lv  Std.all
    mast              1.000                               1.000    1.000

R-Square:
                   Estimate
    RWI               0.524
    mast              0.652
```

SE2

```
                                                  Used       Total
  Number of observations                           103         115

  Estimator                                       DWLS
  Minimum Function Test Statistic                1.489
  Degrees of freedom                                 5
  P-value (Chi-square)                           0.914

Model test baseline model:

  Minimum Function Test Statistic               85.804
  Degrees of freedom                                11
  P-value                                        0.000

User model versus baseline model:

  Comparative Fit Index (CFI)                    1.000
  Tucker-Lewis Index (TLI)                       1.103

Root Mean Square Error of Approximation:

  RMSEA                                          0.000
  90 Percent Confidence Interval          0.000  0.051
  P-value RMSEA <= 0.05                          0.949

Standardized Root Mean Square Residual:

  SRMR                                           0.015

Weighted Root Mean Square Residual:

  WRMR                                           0.326

Parameter Estimates:

  Information                                 Expected
  Standard Errors                             Standard

Regressions:
                   Estimate  Std.Err  z-value  P(>|z|)   Std.lv  Std.all
  RWI ~
    PRE               0.224    0.096    2.340    0.019    0.224    0.219
    MAX              -0.131    0.103   -1.269    0.204   -0.131   -0.131
    mast             -0.297    0.063   -4.709    0.000   -0.297   -0.567
    RWI.lag1          0.348    0.096    3.616    0.000    0.348    0.358
  mast ~
    MAX.L             1.038    0.198    5.251    0.000    1.038    0.534
    MAX.LL           -1.121    0.238   -4.709    0.000   -1.121   -0.604

Intercepts:
                   Estimate  Std.Err  z-value  P(>|z|)   Std.lv  Std.all
   .RWI              -0.021    0.088   -0.240    0.810   -0.021   -0.021
   .mast              0.000                               0.000    0.000

Thresholds:
                   Estimate  Std.Err  z-value  P(>|z|)   Std.lv  Std.all
    mast|t1           0.802    0.221    3.631    0.000    0.802    0.425

Variances:
                   Estimate  Std.Err  z-value  P(>|z|)   Std.lv  Std.all
   .RWI               0.561    0.094    5.998    0.000    0.561    0.574
   .mast              1.000                               1.000    0.281

Scales y*:
                   Estimate  Std.Err  z-value  P(>|z|)   Std.lv  Std.all
    mast              1.000                               1.000    1.000

R-Square:
                   Estimate
    RWI               0.426
    mast              0.719
```

DE1

```
                                                  Used       Total
  Number of observations                           108         115

  Estimator                                       DWLS
  Minimum Function Test Statistic                2.920
  Degrees of freedom                                 5
  P-value (Chi-square)                           0.712

Model test baseline model:

  Minimum Function Test Statistic               57.475
  Degrees of freedom                                11
  P-value                                        0.000

User model versus baseline model:

  Comparative Fit Index (CFI)                    1.000
  Tucker-Lewis Index (TLI)                       1.098

Root Mean Square Error of Approximation:

  RMSEA                                          0.000
  90 Percent Confidence Interval          0.000  0.100
  P-value RMSEA <= 0.05                          0.812

Standardized Root Mean Square Residual:

  SRMR                                           0.081

Weighted Root Mean Square Residual:

  WRMR                                           0.457

Parameter Estimates:

  Information                                 Expected
  Standard Errors                             Standard

Regressions:
                   Estimate  Std.Err  z-value  P(>|z|)   Std.lv  Std.all
  RWI ~
    PRE               0.497    0.118    4.224    0.000    0.497    0.507
    MAX              -0.136    0.092   -1.488    0.137   -0.136   -0.138
    mast             -0.053    0.053   -1.011    0.312   -0.053   -0.090
    RWI.lag1          0.256    0.119    2.163    0.031    0.256    0.256
  mast ~
    MAX.L             0.861    0.275    3.136    0.002    0.861    0.514
    MAX.LL           -1.159    0.271   -4.275    0.000   -1.159   -0.692

Intercepts:
                   Estimate  Std.Err  z-value  P(>|z|)   Std.lv  Std.all
   .RWI               0.007    0.082    0.091    0.928    0.007    0.007
   .mast              0.000                               0.000    0.000

Thresholds:
                   Estimate  Std.Err  z-value  P(>|z|)   Std.lv  Std.all
    mast|t1           0.891    0.209    4.256    0.000    0.891    0.531

Variances:
                   Estimate  Std.Err  z-value  P(>|z|)   Std.lv  Std.all
   .RWI               0.638    0.077    8.324    0.000    0.638    0.639
   .mast              1.000                               1.000    0.355

Scales y*:
                   Estimate  Std.Err  z-value  P(>|z|)   Std.lv  Std.all
    mast              1.000                               1.000    1.000

R-Square:
                   Estimate
    RWI               0.361
    mast              0.645
```

DE2-low

```
                                                  Used       Total
  Number of observations                           111         115

  Estimator                                       DWLS
  Minimum Function Test Statistic                0.896
  Degrees of freedom                                 5
  P-value (Chi-square)                           0.971

Model test baseline model:

  Minimum Function Test Statistic               30.463
  Degrees of freedom                                11
  P-value                                        0.001

User model versus baseline model:

  Comparative Fit Index (CFI)                    1.000
  Tucker-Lewis Index (TLI)                       1.464

Root Mean Square Error of Approximation:

  RMSEA                                          0.000
  90 Percent Confidence Interval          0.000  0.000
  P-value RMSEA <= 0.05                          0.984

Standardized Root Mean Square Residual:

  SRMR                                           0.015

Weighted Root Mean Square Residual:

  WRMR                                           0.253

Parameter Estimates:

  Information                                 Expected
  Standard Errors                             Standard

Regressions:
                   Estimate  Std.Err  z-value  P(>|z|)   Std.lv  Std.all
  RWI ~
    PRE               0.215    0.126    1.704    0.088    0.215    0.215
    MAX              -0.176    0.110   -1.597    0.110   -0.176   -0.175
    mast             -0.215    0.080   -2.695    0.007   -0.215   -0.266
    RWI.lag1          0.126    0.111    1.128    0.259    0.126    0.123
  mast ~
    MAX.L             0.505    0.147    3.441    0.001    0.505    0.402
    MAX.LL           -0.632    0.315   -2.007    0.045   -0.632   -0.503

Intercepts:
                   Estimate  Std.Err  z-value  P(>|z|)   Std.lv  Std.all
   .RWI               0.004    0.096    0.041    0.967    0.004    0.004
   .mast              0.000                               0.000    0.000

Thresholds:
                   Estimate  Std.Err  z-value  P(>|z|)   Std.lv  Std.all
    mast|t1           1.563    0.318    4.915    0.000    1.563    1.248

Variances:
                   Estimate  Std.Err  z-value  P(>|z|)   Std.lv  Std.all
   .RWI               0.813    0.104    7.806    0.000    0.813    0.799
   .mast              1.000                               1.000    0.637

Scales y*:
                   Estimate  Std.Err  z-value  P(>|z|)   Std.lv  Std.all
    mast              1.000                               1.000    1.000

R-Square:
                   Estimate
    RWI               0.201
    mast              0.363
```

DE2-high

```
                                                  Used       Total
  Number of observations                           109         115

  Estimator                                       DWLS
  Minimum Function Test Statistic                5.869
  Degrees of freedom                                 5
  P-value (Chi-square)                           0.319

Model test baseline model:

  Minimum Function Test Statistic               42.611
  Degrees of freedom                                11
  P-value                                        0.000

User model versus baseline model:

  Comparative Fit Index (CFI)                    0.973
  Tucker-Lewis Index (TLI)                       0.940

Root Mean Square Error of Approximation:

  RMSEA                                          0.040
  90 Percent Confidence Interval          0.000  0.144
  P-value RMSEA <= 0.05                          0.469

Standardized Root Mean Square Residual:

  SRMR                                           0.027

Weighted Root Mean Square Residual:

  WRMR                                           0.647

Parameter Estimates:

  Information                                 Expected
  Standard Errors                             Standard

Regressions:
                   Estimate  Std.Err  z-value  P(>|z|)   Std.lv  Std.all
  RWI ~
    PRE              -0.109    0.117   -0.928    0.354   -0.109   -0.111
    MAX               0.170    0.115    1.480    0.139    0.170    0.171
    mast             -0.156    0.080   -1.956    0.050   -0.156   -0.203
    RWI.lag1          0.331    0.086    3.871    0.000    0.331    0.320
  mast ~
    MAX.L             0.742    0.207    3.576    0.000    0.742    0.570
    MAX.LL           -0.496    0.332   -1.493    0.136   -0.496   -0.378

Intercepts:
                   Estimate  Std.Err  z-value  P(>|z|)   Std.lv  Std.all
   .RWI              -0.015    0.101   -0.149    0.882   -0.015   -0.015
   .mast              0.000                               0.000    0.000

Thresholds:
                   Estimate  Std.Err  z-value  P(>|z|)   Std.lv  Std.all
    mast|t1           1.661    0.348    4.776    0.000    1.661    1.275

Variances:
                   Estimate  Std.Err  z-value  P(>|z|)   Std.lv  Std.all
   .RWI               0.805    0.126    6.386    0.000    0.805    0.809
   .mast              1.000                               1.000    0.589

Scales y*:
                   Estimate  Std.Err  z-value  P(>|z|)   Std.lv  Std.all
    mast              1.000                               1.000    1.000

R-Square:
                   Estimate
    RWI               0.191
    mast              0.411
```

Appendix S8

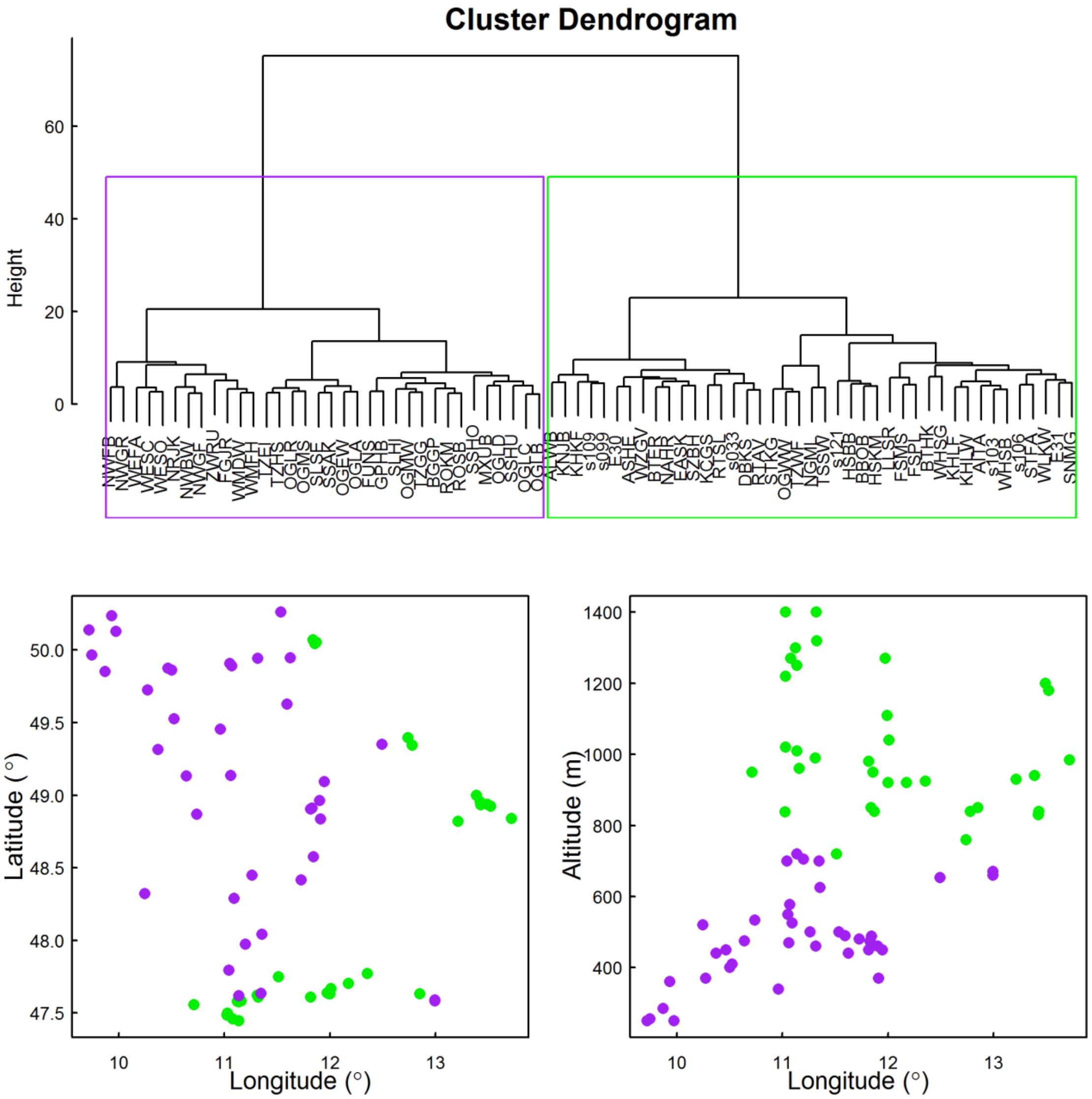

Cluster analysis of the DE2 chronologies, and the two main groups mapped and plotted against elevation. Purple = DE2-low, Green = DE2-high. Chronologies were analysed using Wards Hierarchical clustering, with Euclidean distances. Analysis was restricted to the period of common overlap between all chronologies.

Appendix S9

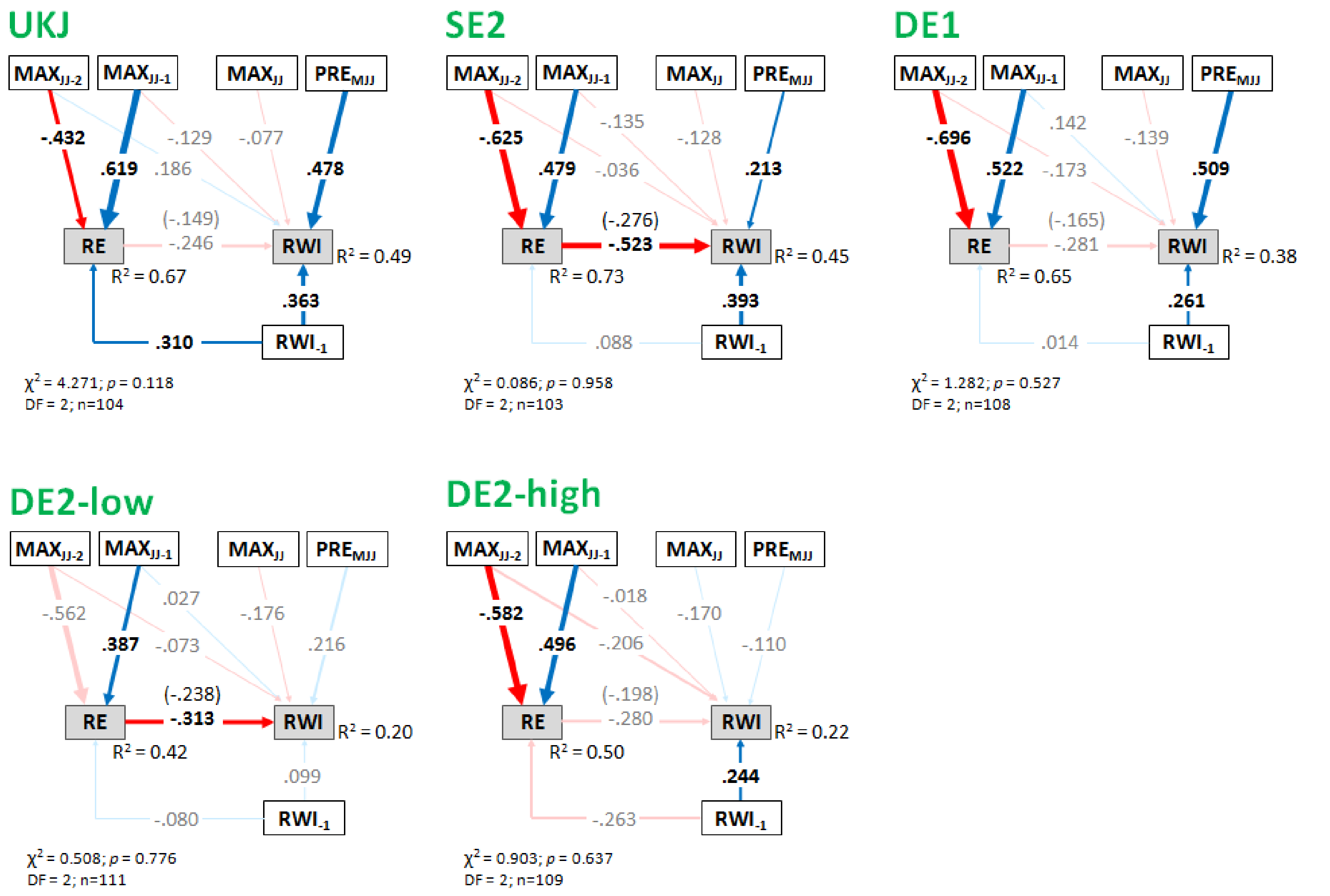

Structural Equation Models/Path analysis diagrams representing the effects of temperature and precipitation on radial growth, with indirect pathways involving the effects of allocation to reproduction (*RE*). The model structure presented here corresponds to the full theoretical model presented in Figure 1. Blue and red arrows indicate positive and negative relationships, respectively. Numbers on the arrows indicate the standardized path coefficients, with arrow thickness proportional to the coefficient strength. Pale colours indicate non-significant pathways (p<0.05). The proportion of explained variance (R^2) for each endogenous variable is also shown.

Appendix S10

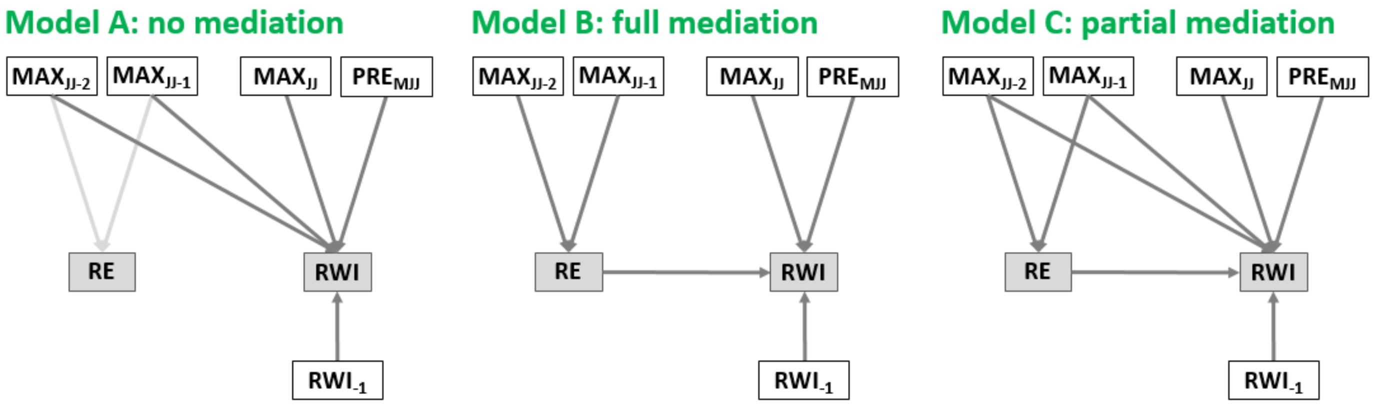

Models based on alternative linkages between previous summers' temperature and growth.

Model A: no mediation model. Model A describes the situation where RWI is modelled only as a function of climate variables and previous year RWI; i.e. masting is not a predictor of tree growth, but previous summers' temperature influence growth directly

Model B: full mediation model. Model B describes the situation where RWI is modelled only as a function of growing climate variables, previous year RWI and masting. Previous summers' temperature can only influence RWI indirectly, through their effect on mast.

Model C: partial mediation model. Model C describes the situation where RWI is modelled as a function of climate variables (in the year of growth and previous years), previous year RWI and masting; i.e. previous summers' temperature can influence RWI directly and indirectly through mast. Model C therefore represents a combination of Model A and B.

Mediation analysis is used as a formal comparison of the performance of these three difference models for each of the five chronologies analysed.

Mediation analysis

**SE2**	R^2_{RWI}	X^2	X^2 diff	p-value
C (Partial mediation)	0.441	0.9308		
A (No mediation)	0.370	10.7144	9.7836	0.0018*
B (Full mediation)	0.425	1.5368	-9.1776	1.0000
**UKJ**	R^2_{RWI}	X^2	X^2 diff	p-value
C (Partial mediation)	0.489	8.7415		
A (No mediation)	0.467	10.4375	1.69591	0.1928
B (Full mediation)	0.524	10.6764	0.23891	0.6250
**DE1**	R^2_{RWI}	X^2	X^2 diff	p-value
C (Partial mediation)	0.382	1.2959		
A (No mediation)	0.354	3.8835	2.58756	0.1077
B (Full mediation)	0.361	2.9200	-0.96348	1.0000
**DE2 - high**	R^2_{RWI}	X^2	X^2 diff	p-value
C (Partial mediation)	0.215	2.9706		
A (No mediation)	0.176	5.9652	2.99454	0.0836
B (Full mediation)	0.191	5.8688	-0.09638	1.0000
**DE2 - low**	R^2_{RWI}	X^2	X^2 diff	p-value
C (Partial mediation)	0.204	0.6268		
A (No mediation)	0.147	0.70732	6.4464	0.0111*
B (Full mediation)	0.201	0.8958	-6.1775	1.0000

In all five cases, the model C and B are statistically indistinguishable, and including the direct links between previous summers' temperature and RWI does not increase model performance.

Additionally, in SE2 and DE2-low, the model where RWI is modelled as a function of climate and previous years RWI (and not RE) performs significantly worse than models that do include RE. In DE2-high, this improvement is marginally insignificant.

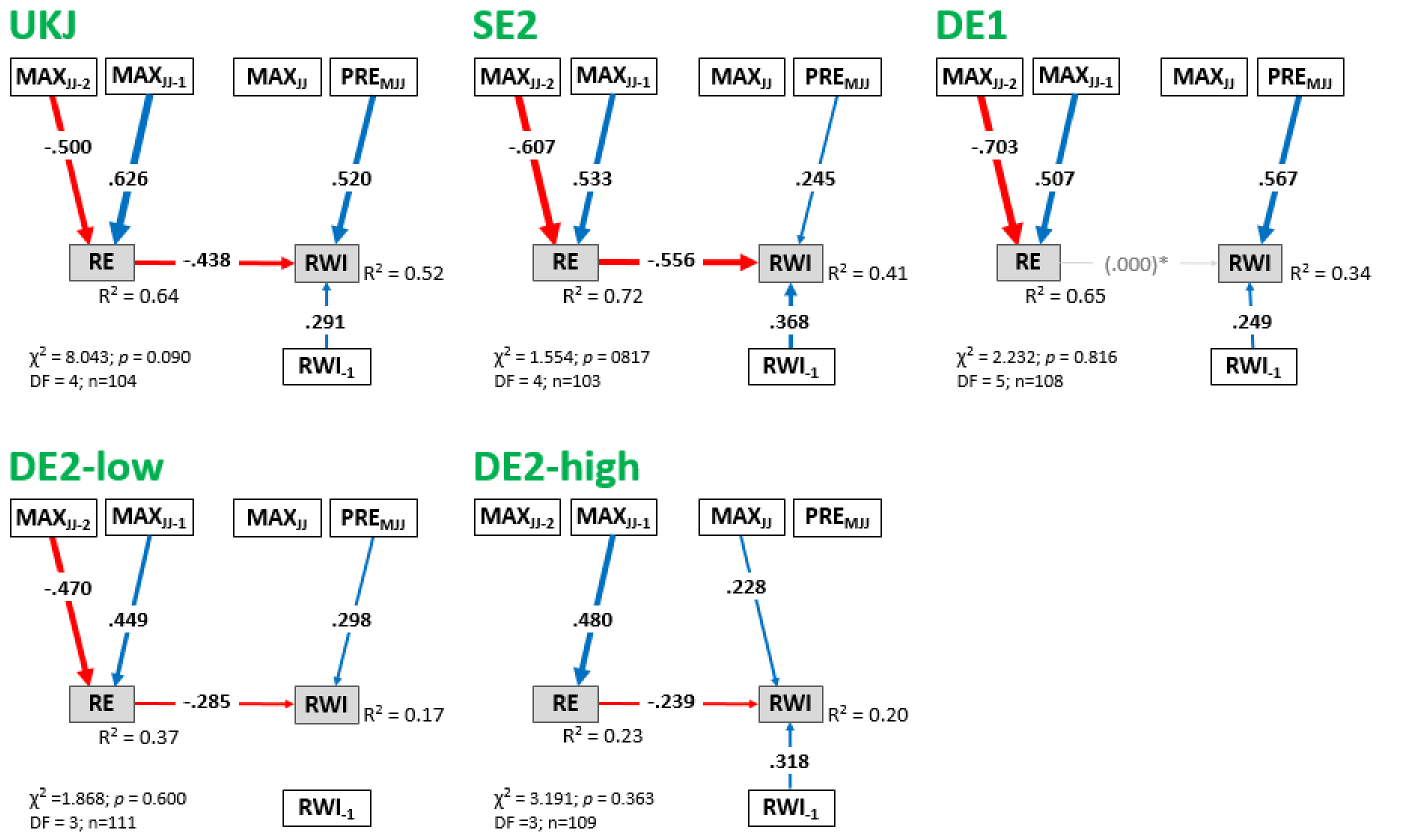

Figure 3c: Structural Equation Models/Path analysis diagrams representing the effects of temperature and precipitation on radial growth, with indirect pathways involving the effects of allocation to reproduction (*RE*). Following mediation analysis, direct pathways from MAX_{JJ-1} and MAX_{JJ-2} to RWI, and from RWI_{-1} to *RE*, have been removed. Additional insignificant linkages are also removed from individual models, unless they significantly reduce the model GOF. Blue and red arrows indicate positive and negative relationships, respectively. Numbers on the arrows indicate the standardized path coefficients, with arrow thickness proportional to the coefficient strength. Pale colours indicate non-significant pathways (p<0.05). The proportion of explained variance (R^2) for each endogenous variable is also shown.

Appendix S12

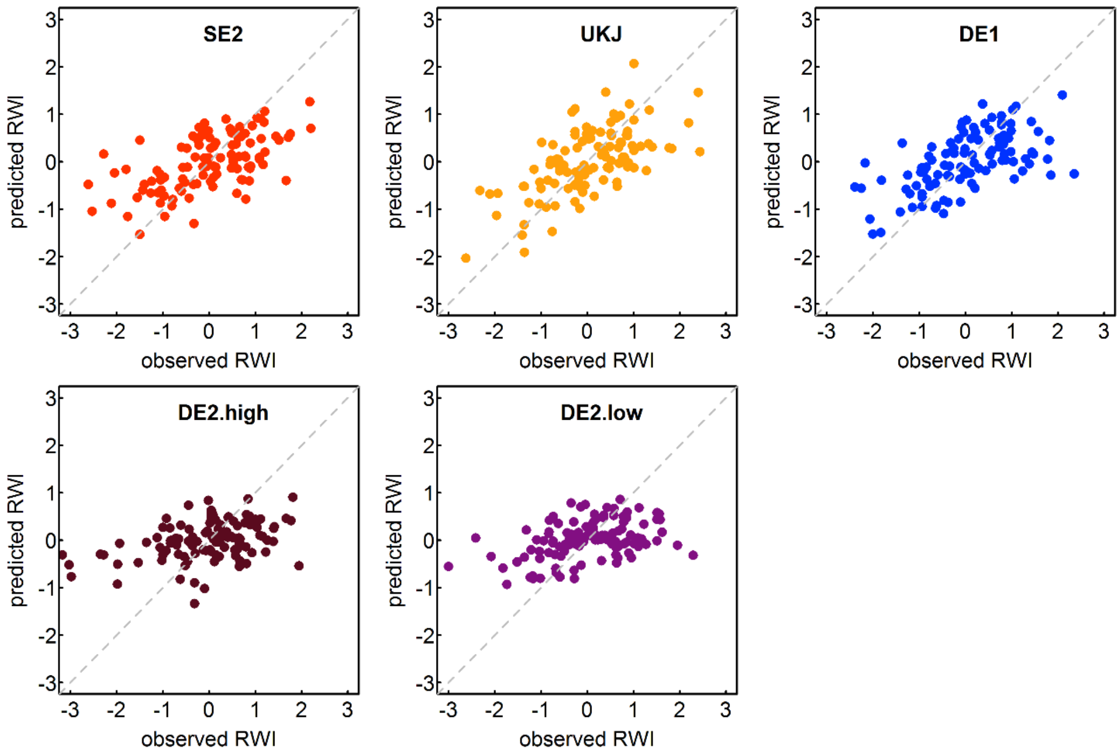

Scatterplots of observed and predicted RWI for the five model development regions, corresponding to the data presented in Figure 4.

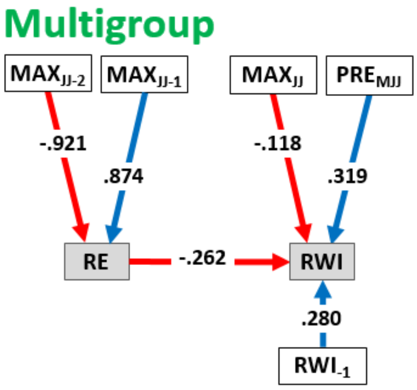

Multi-group model used for the prediction of RWI in 16 independent regions (Figure 6). Raw coefficients are plotted, as used in the predictive model.

```
    Number of observations per group
  UKJ                                                104
  SE2                                                103
  DE2.1                                              111

  Estimator                                         DWLS
  Minimum Function Test Statistic                 42.305
  Degrees of freedom                                  31
  P-value (Chi-square)                             0.085

Chi-square for each group:

  UKJ                                             17.583
  SE2                                              7.867
  DE2.1                                           16.855

Parameter Estimates:

  Information                                   Expected
  Standard Errors                               Standard


Group 1 [UKJ]:

Regressions:
                   Estimate  Std.Err  z-value  P(>|z|)   Std.lv  Std.all
  RWI ~
    PRE     (.p1.)    0.319    0.061    5.212    0.000    0.319    0.319
    mast    (.p2.)   -0.262    0.038   -6.894    0.000   -0.262   -0.421
    RWI.lg1 (.p3.)    0.280    0.052    5.365    0.000    0.280    0.285
    MAX     (.p4.)   -0.118    0.055   -2.137    0.033   -0.118   -0.119
  mast ~
    MAX.L   (.p5.)    0.874    0.131    6.663    0.000    0.874    0.528
    MAX.LL  (.p6.)   -0.921    0.150   -6.136    0.000   -0.921   -0.576

Intercepts:
                   Estimate  Std.Err  z-value  P(>|z|)   Std.lv  Std.all
   .RWI     (.11.)   -0.011    0.051   -0.222    0.825   -0.011   -0.011
   .mast              0.000                               0.000    0.000

Thresholds:
                   Estimate  Std.Err  z-value  P(>|z|)   Std.lv  Std.all
    mast|t1 (.p7.)    1.091    0.179    6.099    0.000    1.091    0.687

Variances:
                   Estimate  Std.Err  z-value  P(>|z|)   Std.lv  Std.all
   .RWI     (.p8.)    0.607    0.055   10.982    0.000    0.607    0.624
   .mast              1.000                               1.000    0.396

Scales y*:
                   Estimate  Std.Err  z-value  P(>|z|)   Std.lv  Std.all
    mast              1.000                               1.000    1.000

R-Square:
                   Estimate
    RWI               0.376
    mast              0.604
```

```
Group 2 [SE2]:

Regressions:
                   Estimate  Std.Err  z-value  P(>|z|)   Std.lv  Std.all
  RWI ~
    PRE     (.p1.)    0.319    0.061    5.212    0.000    0.319    0.318
    mast    (.p2.)   -0.262    0.038   -6.894    0.000   -0.262   -0.447
    RWI.lg1 (.p3.)    0.280    0.052    5.365    0.000    0.280    0.293
    MAX     (.p4.)   -0.118    0.055   -2.137    0.033   -0.118   -0.121
  mast ~
    MAX.L   (.p5.)    0.874    0.131    6.663    0.000    0.874    0.510
    MAX.LL  (.p6.)   -0.921    0.150   -6.136    0.000   -0.921   -0.563

Intercepts:
                   Estimate  Std.Err  z-value  P(>|z|)   Std.lv  Std.all
   .RWI     (.11.)   -0.011    0.051   -0.222    0.825   -0.011   -0.012
   .mast              0.000                                0.000    0.000

Thresholds:
                   Estimate  Std.Err  z-value  P(>|z|)   Std.lv  Std.all
    mast|t1 (.p7.)    1.091    0.179    6.099    0.000    1.091    0.656

Variances:
                   Estimate  Std.Err  z-value  P(>|z|)   Std.lv  Std.all
   .RWI     (.p8.)    0.607    0.055   10.982    0.000    0.607    0.641
   .mast              1.003                                1.003    0.362

Scales y*:
                   Estimate  Std.Err  z-value  P(>|z|)   Std.lv  Std.all
    mast              0.999    0.160    6.250    0.000    0.999    1.000

R-Square:
                   Estimate
    RWI               0.359
    mast              0.638


Group 3 [DE2.l]:

Regressions:
                   Estimate  Std.Err  z-value  P(>|z|)   Std.lv  Std.all
  RWI ~
    PRE     (.p1.)    0.319    0.061    5.212    0.000    0.319    0.315
    mast    (.p2.)   -0.262    0.038   -6.894    0.000   -0.262   -0.434
    RWI.lg1 (.p3.)    0.280    0.052    5.365    0.000    0.280    0.271
    MAX     (.p4.)   -0.118    0.055   -2.137    0.033   -0.118   -0.116
  mast ~
    MAX.L   (.p5.)    0.874    0.131    6.663    0.000    0.874    0.513
    MAX.LL  (.p6.)   -0.921    0.150   -6.136    0.000   -0.921   -0.541

Intercepts:
                   Estimate  Std.Err  z-value  P(>|z|)   Std.lv  Std.all
   .RWI     (.11.)   -0.011    0.051   -0.222    0.825   -0.011   -0.011
   .mast              0.000                                0.000    0.000

Thresholds:
                   Estimate  Std.Err  z-value  P(>|z|)   Std.lv  Std.all
    mast|t1 (.p7.)    1.091    0.179    6.099    0.000    1.091    0.643

Variances:
                   Estimate  Std.Err  z-value  P(>|z|)   Std.lv  Std.all
   .RWI     (.p8.)    0.607    0.055   10.982    0.000    0.607    0.580
   .mast              1.477                                1.477    0.514

Scales y*:
                   Estimate  Std.Err  z-value  P(>|z|)   Std.lv  Std.all
    mast              0.823    0.143    5.759    0.000    0.823    1.000

R-Square:
                   Estimate
    RWI               0.420
    mast              0.486
```

Fit parameters for multi-group model

npar	fmin	chisq	df
11.000	0.067	42.305	31.000
pvalue	baseline.chisq	baseline.df	baseline.pvalue
0.085	217.888	33.000	0.000
cfi	tli	nnfi	rfi
0.939	0.935	0.935	0.793
nfi	pnfi	ifi	rni
0.806	0.757	0.940	0.939
rmsea	rmsea.ci.lower	rmsea.ci.upper	rmsea.pvalue
0.059	0.000	0.100	0.346
rmr	rmr_nomean	srmr	srmr_bentler
0.054	0.069	0.095	0.074
srmr_bentler_nomean	srmr_bollen	srmr_bollen_nomean	srmr_mplus
0.095	0.040	0.050	0.074
srmr_mplus_nomean	cn_05	cn_01	gfi
0.095	335.955	389.610	0.906
agfi	pgfi	mfi	
0.872	0.668	0.982	

Appendix S14

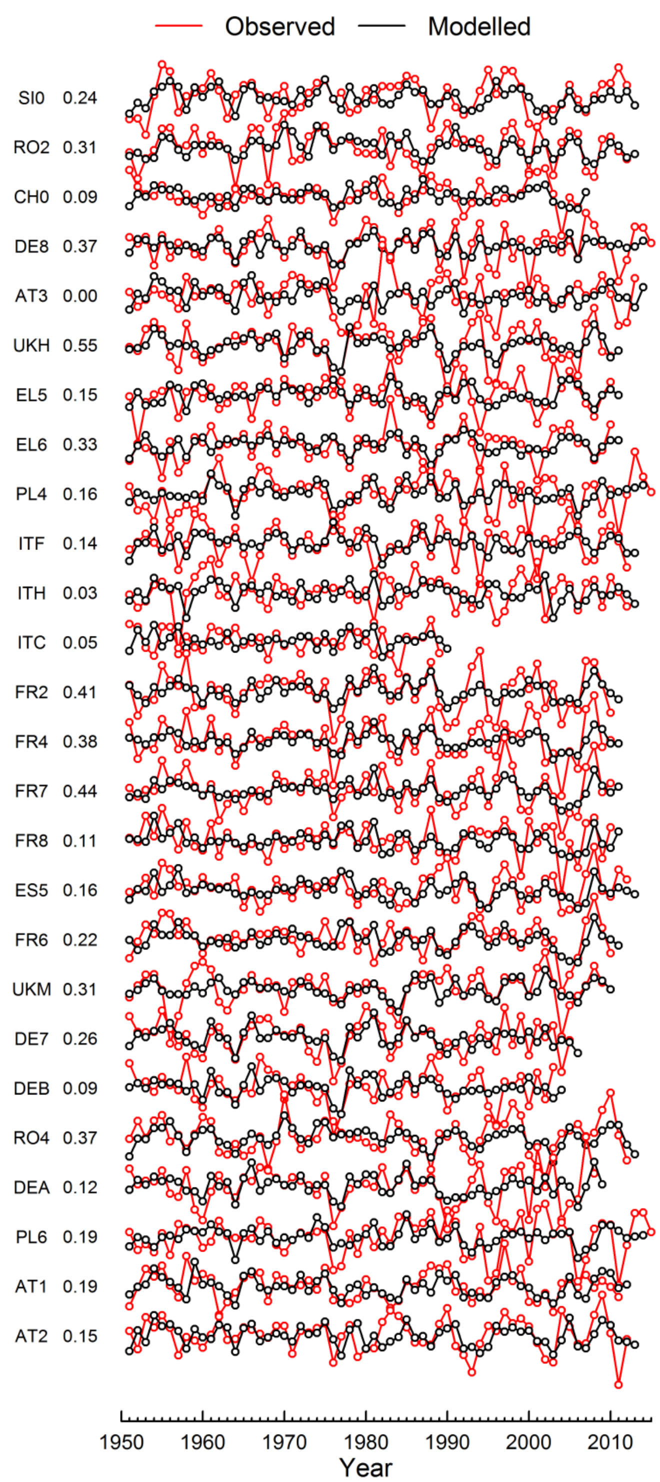

Comparison of observed and predicted RWI for independent datasets. Printed value is the R^2 between observed and predicted values. Note that in these models, mast years are predicted using the climate data (i.e. mast data is not used in the prediction of RWI).

Appendix S15

As data for growth (RWI) was detrended, we repeated analysis with identically detrended climate data, revealing no major changes in model structure or the values of parameters, and no change in conclusions.

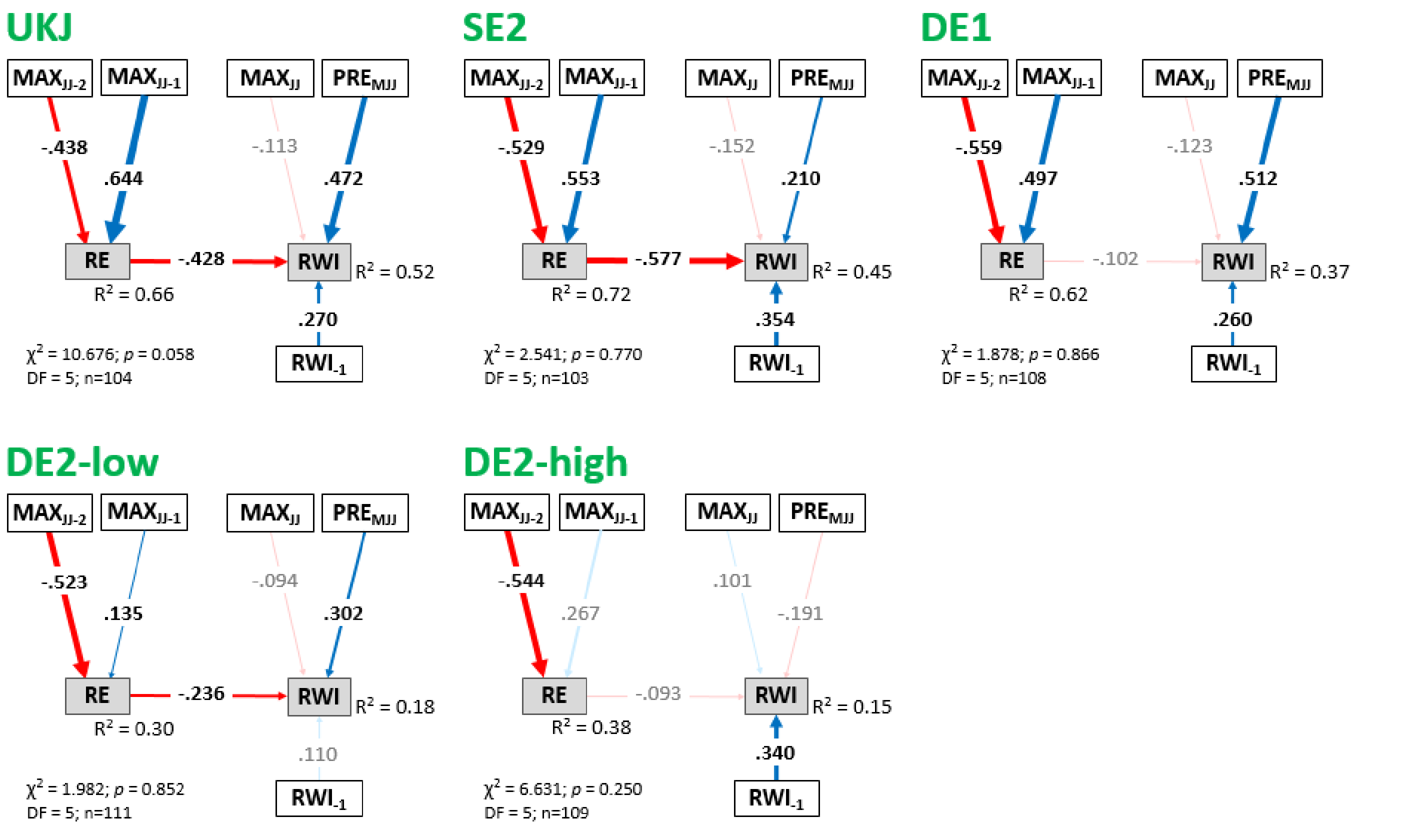

Supplement: Supplementary file 1 [file ELE-21-1833-s001.pdf]
